# Transcriptional profiling identifies strain-specific effects of caloric restriction and opposite responses in human and mouse white adipose tissue

**DOI:** 10.18632/aging.101424

**Published:** 2018-04-29

**Authors:** William R. Swindell, Edward O. List, Darlene E. Berryman, John J. Kopchick

**Affiliations:** 1 Heritage College of Osteopathic Medicine, Ohio University, Athens, OH 45701, USA; 2 Edison Biotechnology Institute, Ohio University, Athens, OH 45701, USA; 3 The Diabetes Institute, Ohio University, Athens, OH 45701, USA

**Keywords:** adipose, aging, dietary restriction, insulin, insulin-like growth factor, longevity, microarray, olfactory receptor

## Abstract

Caloric restriction (CR) has been extensively studied in rodents as an intervention to improve lifespan and healthspan. However, effects of CR can be strain- and species-specific. This study used publically available microarray data to analyze expression responses to CR in males from 7 mouse strains (C57BL/6J, BALB/c, C3H, 129, CBA, DBA, B6C3F1) and 4 tissues (epididymal white adipose tissue (eWAT), muscle, heart, cortex). In each tissue, the largest number of strain-specific CR responses was identified with respect to the C57BL/6 strain. In heart and cortex, CR responses in C57BL/6 mice were negatively correlated with responses in other strains. Strain-specific CR responses involved genes associated with olfactory receptors (*Olfr1184*, *Olfr910*) and insulin/IGF-1 signaling (*Igf1*, *Irs2*). In each strain, CR responses in eWAT were negatively correlated with those in human subcutaneous WAT (scWAT). In human scWAT, CR increased expression of genes associated with stem cell maintenance and vascularization. However, orthologous genes linked to these processes were down-regulated in mouse. These results identify strain-specific CR responses limiting generalization across mouse strains. Differential CR responses in mouse versus human WAT may be due to differences in the depots examined and/or the presence of “thrifty genes” in humans that resist adipose breakdown despite caloric deficit.

## Introduction

Caloric restriction (CR) has been extensively studied as an intervention hypothesized to lengthen healthspan, delay age-related disease and promote longevity. These effects have been supported by observations from a broad range of invertebrate and vertebrate organisms [1], although foundational experiments demonstrating favorable effects of CR on aging were performed using rodents [2,3]. The idea that CR improves mouse lifespan has for decades served as a guiding assumption in experimental aging research [4]. In recent years, however, an increasingly nuanced CR paradigm has emerged with greater recognition of murine genetic factors [5], based upon the accretion of evidence demonstrating diverse responses to CR across inbred mouse strains [6], failure of CR to improve mean lifespan of wild-derived mice [7], and variable effects of CR on the lifespan of mice from recombinant inbred strain panels [8]. These observations have challenged longstanding ideas regarding CR and its effects on aging, but are not unexpected considering the extensive genetic diversity among inbred mouse strains [9] and corresponding phenotypic differences related to disease propensity [10], body composition [11] and circulating hormone levels [12]. Nonetheless, mouse strain differences complicate studies in aging and other fields of experimental medicine because mechanistic conclusions established from one strain may not be generalizable [13,14]. This diminishes repeatability of research results and challenges efforts to translate findings [15,16], since it is unclear which mouse strains can most faithfully represent the physiology of aging in primate species such as humans [17,18].

The C57BL/6 strain has historically been chosen as a favored background by laboratories studying the effects of CR on mouse aging [19]. The C57BL/6 strain has practical advantages as a reliable breeder with good reproductive lifespan and litter sizes. However, the widespread adoption of C57BL/6 mice as an experimental model is partly due to convention, with many investigators utilizing C57BL/6 mice only to achieve consistency with prior work or concurrent studies in other laboratories. C57BL/6 mice are indeed susceptible to age-related conditions exacerbated by high-calorie diets, such as obesity, type 2 diabetes and atherosclerosis [10], which may explain why they often respond favorably to CR diets, with one meta-analysis reporting that CR-fed C57BL/6 mice live 6.7% longer on average compared to *ad lib*-fed mice (*n* = 22 experiments) [20]. However, responses of C57BL/6 mice to CR differ in comparison to other strains that do not reliably demonstrate improved longevity when provided a CR diet (e.g., DBA/2 mice) [21–23]. Compared to DBA/2 mice, for example, CR-fed C57BL/6 mice demonstrate stronger or more rapid improvements in glucose tolerance [21], cellular redox status [22], and skeletal muscle cell progenitor abundance [23]. More importantly, the response of mouse strains to CR may depend upon metabolic factors determining adipose mass during the course of aging [24–26]. The C57BL/6 strain, for example, appears to retain fat mass with aging better than DBA/2 mice [26], and along the same lines recombinant inbred strains responding favorably to CR are better able to maintain adiposity compared to those strains with lifespan shortening due to CR [25].

Given differences in CR responses among mouse strains, the question arises of which strain(s) can provide the best models for biomedical translation to humans [20,27]. The most commonly studied mouse strains, such as C57BL/6, may not faithfully replicate human CR responses or may otherwise be misleading. For instance, CR decreases circulating IGF-1 levels in C57BL/6 mice [21,28], but in contrast CR increases circulating IGF-1 in humans [29,30]. More broadly, some investigators have expressed skepticism about whether any mouse strain can be useful for modeling human dietary responses, citing species divergence of dietary preferences, differences in feeding behavior, artificial aspects of the rodent laboratory environment, and fundamental life history differences of rodents as compared to humans [31,32]. These disparities may diminish the predictive validity of mouse models in dietary research on aging, and increasingly investigators are encouraged to provide substantive validation to support mouse models for translational purposes [33]. Along these lines, expression profiling of mRNA abundance using microarray or RNA-seq provides a quantitative strategy for comparing CR responses with respect to orthologous genes, and this approach has been applied in other contexts to score the strengths and weakness of mouse phenotypes in terms of resemblance to human diseases [34–36]. Moreover, given CR response data from multiple mouse strains, transcriptome-based analyses can be used to discriminate among different mouse strains and identify those that most faithfully recapitulate human CR responses [35].

The goals of this study were to apply transcriptomics to identify strain-specific CR responses in the laboratory mouse and to perform a strain-by-strain comparison to CR responses observed in a corresponding human tissue. We therefore used a recently published microarray dataset [37] to evaluate gene expression responses to CR in 4 tissues (epididymal white adipose tissue [eWAT], muscle, heart, cortex) from males of 7 mouse strains (C57BL/6J [B6], Balbc/J, C3H/HeJ, 129S1/SvImJ, CBA/J, DBA/2J, B6C3F1/J [F1]). Utilizing techniques for dimensionality reduction and multivariate analysis, our results provide a comparison of CR responses across 28 strain-tissue combinations (7 strains × 4 tissues). We further perform a meta-analysis of human studies that have evaluated subcutaneous WAT (scWAT) transcriptome responses to CR [38-46], allowing us to extract a robust meta-signature of CR responses in scWAT. This human WAT signature is cross-referenced with CR responses in each mouse strain to identify points of correspondence and non-correspondence at the level of genes and associated biological processes.

## RESULTS

### Tissue and strain impact the number of differentially expressed genes identified in comparisons between CR and control mice

Gene expression data was obtained from a previously published study [37] evaluating the effects of a short-term (14 week) CR diet in males starting at 8 weeks of age. For each of 7 strains, CR-fed mice were provided a diet at least 23% less per week by weight compared to CTL mice (range: 23 – 42%; see Table S1 and Methods). Genes differentially expressed between CR-fed and *ad lib*-fed mice were identified with respect to the 7 mouse strains and 4 tissue types. The number of differentially expressed genes (DEGs) varied among strains and tissues, with no DEGs identified in some strains/tissues but as many as 984 DEGs identified with respect to eWAT from F1 mice (FDR < 0.10, FC > 1.50 or FC < 0.67) (Figures S1 and S2). Effects of CR were strongest in eWAT with the largest number of DEGs identified in eWAT for 5 of 7 strains (exceptions: B6 and BALB/c mice). In eWAT, the largest number of DEGs was identified in F1 mice (984), whereas in muscle and heart most DEGs were identified in C57BL/6 (170 and 557, respectively). No DEGs were identified with respect to cortex except 3 DEGs were increased by CR in CBA mice. Among the 7 strains, the fewest DEGs was usually identified in BALB/c mice and altogether only 13 DEGs were identified in BALB/c mice among the 4 tissues. The magnitude of CR’s effects thus varied considerably among the 28 strain/tissue combinations evaluated.

### Genome-wide transcriptional responses to CR in C57BL/6 mice are weakly correlated with those in other mouse strains (muscle, heart and cortex)

**Figure 1:**
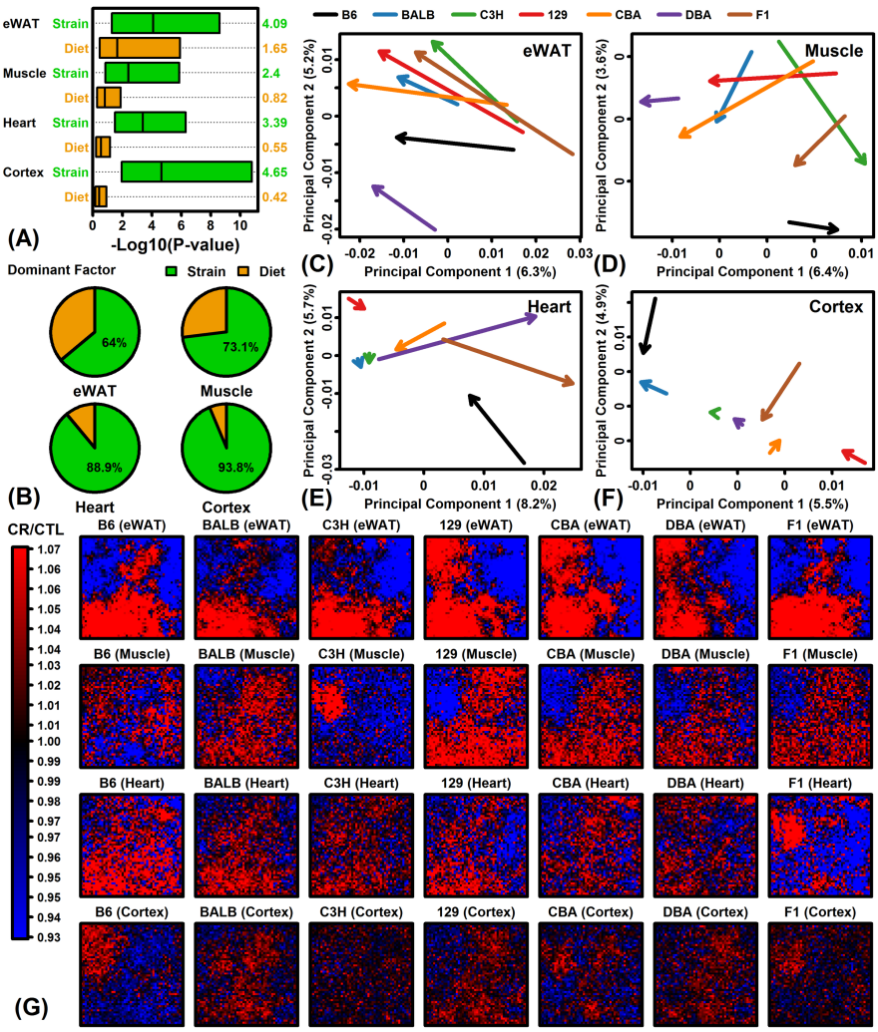
**Global transcriptome responses to CR in 4 tissues (eWAT, muscle, heart, cortex) and 7 mouse strains (B6, BALB/c, C3H, 129, CBA, DBA, F1).** (**A**) Relative importance of strain and diet as factors explaining gene expression variation. Linear models were fit with and without diet and strain as explanatory factors. Factor importance (diet or strain) was evaluated based upon –log10-transformed p-values (Log10P) from likelihood ratio tests applied to each gene (horizontal axis). Bars span the middle 50% of Log10P values among genes (right margin: median Log10P). (**B**) Percentage of genes with strain or diet as dominant explanatory factor. For each gene, the factor yielding the largest Log10P was considered dominant. (**C – F**) Principal component (PC) response vectors. Arrows begin at the bivariate mean of CTL samples and end at the bivariate mean of CR samples. (**G**) Self-organizing maps (SOMs). An SOM layout was determined based upon the expression of 13129 genes with detectable expression in all 4 tissues. Heatmaps show the average FC (CR/CTL) for genes assigned to each SOM region.

Gene expression variation among samples from the same tissue could be explained by strain or diet, and the relative role of these two factors was examined using linear models and likelihood ratio tests. Strain tended to explain a larger fraction of variation in gene expression as compared to diet in all 4 tissues (Figure 1A). Overall, strain was the dominant factor explaining gene expression variation for 64% (eWAT), 73% (muscle), 89% (heart) and 94% (cortex) of protein-coding genes (Figure 1B). The comparatively stronger effect of diet in eWAT could be seen from analysis of CR response vectors in principal component space (Figures 1C – 1F). eWAT response vectors were consistent among strains in terms of length and direction (Figure 1C). However, for muscle, heart and cortex, CR response vectors differed in both length and direction among the 7 strains (Figures 1D – 1F). These patterns were reinforced by visual comparisons of CR response patterns in self-organizing maps (SOMs). In eWAT, CR responses were stronger and more consistent among strains, whereas in muscle, heart and cortex, CR responses were weaker and varied more among strains (Figure 1G).

**Figure 2:**
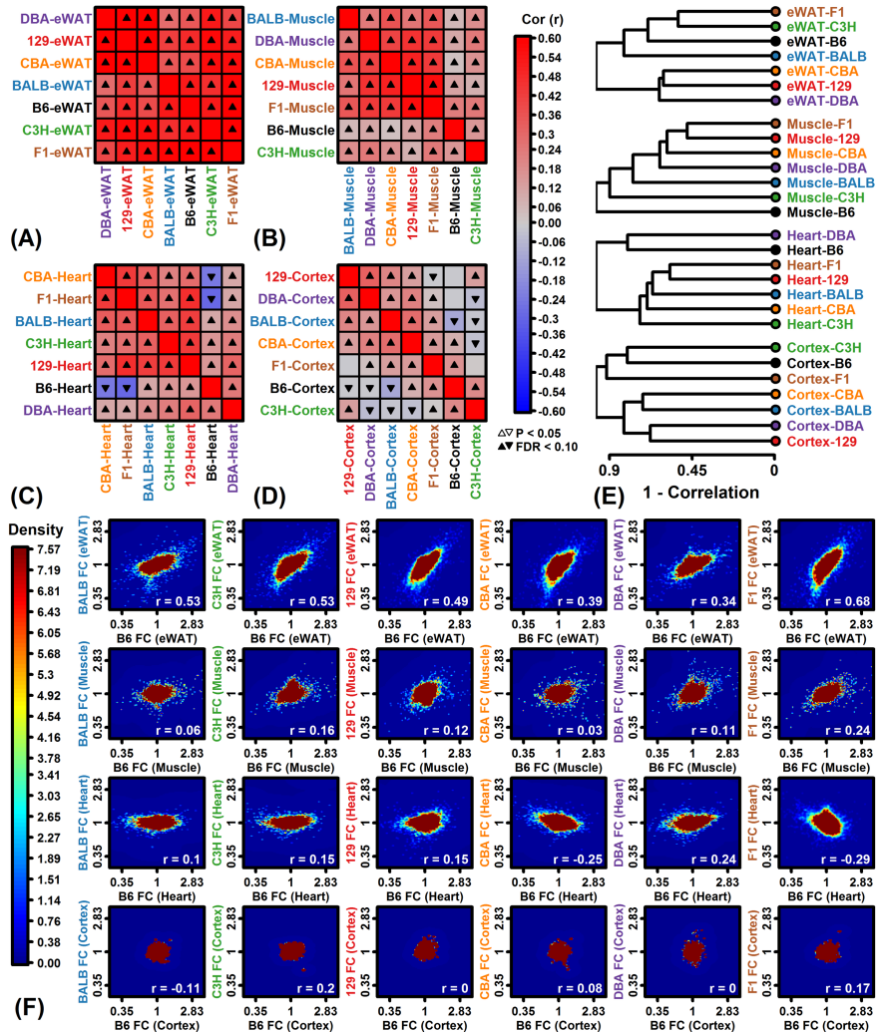
**Genome-wide CR response correlation among strains.** (**A-D**) Spearman rank correlation estimates. (**E**) Hierarchical cluster analysis of strains based upon Spearman correlations among FC estimates. (**F**) Comparison to B6 mice. Scatterplots compare FC estimates in each strain to those from B6 mice. Colors denote gene density (see scale; lower right: Spearman rank correlation). Analyses in (A) – (F) are based upon all protein-coding genes with detectable expression in a given tissue.

The genome-wide correlation between FC estimates among strains was strongest in eWAT but weaker in muscle, heart and cortex (Figures 2A – 2D). In heart and cortex, genome-wide expression responses to CR were negatively correlated for some strain pairs, indicating that many genes were oppositely affected by CR depending on strain (Figures 2C and 2D). Several negative correlation estimates were obtained in pairings that involved the C57BL/6 strain (Figures 2C, 2D and 2F). In heart, for example, the correlation between CR responses in C57BL/6 and F1 mice was -0.29, and likewise, the correlation in C57BL/6 and CBA mice was -0.25 (Figure 2F). When strains were clustered based upon genome-wide correlations among expression responses, the C57BL/6 response was only weakly correlated with that of other strains for muscle, heart and cortex (Figure 2E). For these 3 tissues, expression response correlations between C57BL/6 mice and other strains did not exceed 0.24 (Figure 2F).

**Figure 3:**
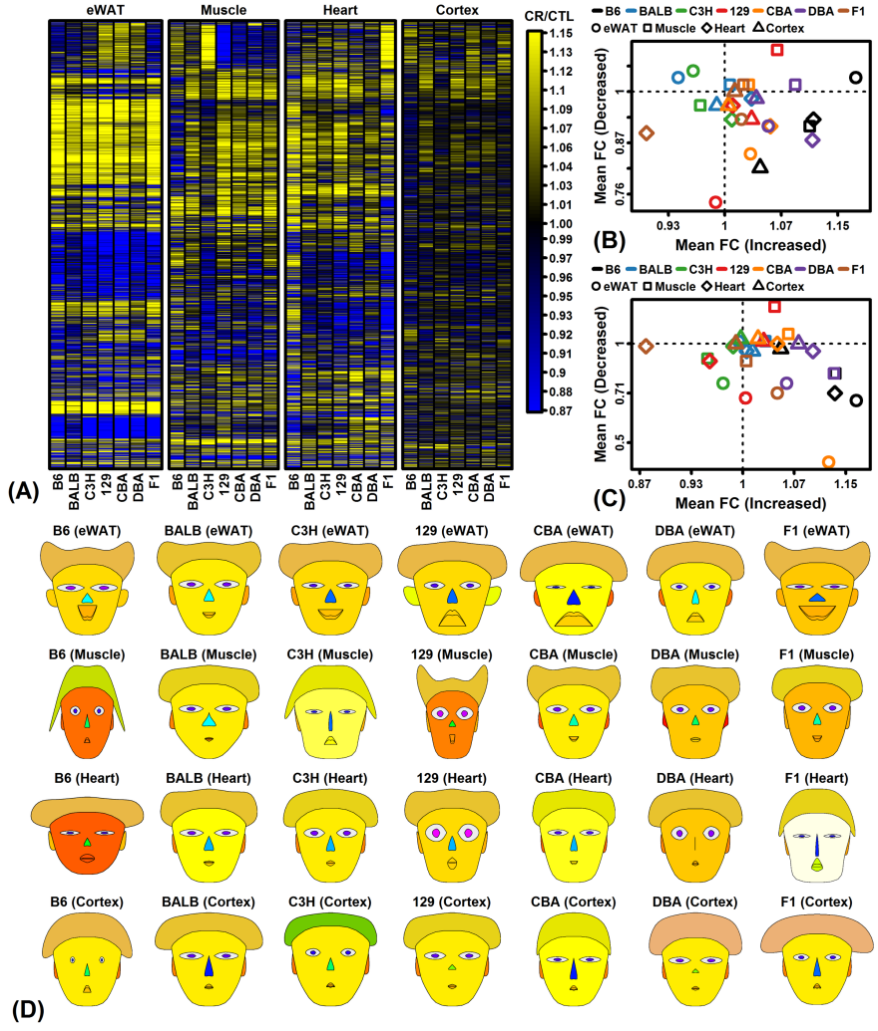
**CR response comparison (28 strain-tissue combinations).** (**A**) FC heatmap with hierarchical clustering. The heatmap shows CR responses among 6564 protein-coding genes with detectable expression in all strains and all 4 tissues. Genes were filtered to exclude those weakly altered by CR. Genes were clustered using the average linkage and Euclidean distance. (**B**) CR meta-analysis gene signature (Plank et al. 2012, Mol Biosyst 8:1339-1349). (**C**) CR meta-analysis gene signature (Swindell 2009, BMC Genomics 10:585). In (B) and (C), average FC is plotted for CR-increased genes (horizontal axis) and CR-decreased genes (vertical axis) identified from meta-analyses of microarray studies of CR response in rodents. Signatures are calculated from (B) 37 CR-increased and 37 CR-decreased genes or (C) 40 CR-increased and 40 CR-decreased genes. (**D**) Chernoff faces. A set of 15 principal component (PC) scores was extracted from the matrix of FC estimates for all protein-coding genes and 28 strain-tissue combinations. The Chernoff face features each correspond to one of the 15 PC scores such that more similar faces indicate more similar CR expression responses.

Side-by-side heatmap comparison of clustered CR responses confirmed better agreement among strains in eWAT, although groups of genes from each tissue could be identified as having strain-specific CR responses (Figure 3A). We analyzed expression of genes previously identified by meta-analysis studies to be similarly altered by CR in multiple strains and tissues [47,48], but found that many strains/tissues did not demonstrate expected trends for such genes (Figures 3B and 3C). For instance, genes expected to be increased by CR were decreased in heart from F1 mice, while genes expected to be decreased by CR were increased in muscle from 129 mice (Figures 3B and 3C). Finally, a summary visual presentation of CR responses was generated using Chernoff faces [49], with facial features scaled and colored according to principal components extracted from a matrix with FC estimates from all expressed protein-coding genes for the 28 strain/tissue combinations (Figure 3D). This multivariate presentation identified strain/tissue combinations with the most aberrant genome-wide CR responses (e.g., B6-muscle, 129-muscle, B6-heart, F1-cortex and C3H-cortex; Figure 3D).

### The number of genes with similar CR responses across strains is larger than expected and primarily involves genes with metabolic functions

Global analysis of gene expression provided evidence for both shared and strain-specific CR responses (Figures 1 – 3). Shared responses to CR among strains or tissues are of special interest, since these may provide the most useful biomarkers or may be conserved among species (and thus replicable in humans) [47,48]. To evaluate shared CR responses, we identified genes similarly altered by CR across strains based upon a nominal p-value threshold of 0.05 per strain (Figure S3). This showed that for all tissues the number of similar CR responses in 6 or all 7 strains was significantly larger than expected under a null hypothesis in which CR responses are randomly associated among strains. A total of 316, 33 and 11 genes were similarly altered by CR in all 7 strains for eWAT, muscle and heart, respectively (P < 0.05 for each strain; Figures S3A – S3C). No genes were similarly altered by CR in cortex from all 7 strains, although a larger-than-expected number were similarly altered by CR in 2 – 6 out of the 7 strains (Figure S3J and S3K). Gene ontology analyses were performed to identify functional themes among those genes most consistently altered by CR across multiple strains (Figure S4). In eWAT, genes increased by CR in all 7 strains were associated with various metabolic functions, while genes decreased by CR in all strains included other metabolic pathways such as gluconeogenesis and ketone metabolism as well as angiogenesis, and response to insulin (Figures S4A and S4B). In other tissues, genes most consistently altered by CR among strains were predominantly associated with metabolism, translation, and/or RNA splicing/metabolism (Figures S4C – S4H).

### C57BL/6 mice have the largest number of strain-specific CR responses including decreased expression of IGF-1 pathway genes in heart

To characterize strain-specific CR responses, linear models were used to identify genes demonstrating strain-by-diet interaction effects, in which responses to CR differed in a given strain as compared to the 6 other strains. In all tissues, the largest number strain-by-diet interaction effects was identified in C57BL/6 mice (Figure S5). Applying an FDR < 0.10 threshold, a total of 256, 833 and 1805 genes exhibited significant interactions in C57BL/6 eWAT, muscle and heart, respectively (Figure S5A – S5D). At this FDR threshold, only 1 gene demonstrated strain-by-diet interaction in cortex of C57BL/6 mice (*Sorcs3*), but 1074 genes were identified at a weaker P < 0.05 threshold, which was a larger number than any of the other 6 strains (Figure S5D).

The largest number of C57BL/6-specific CR responses were identified in heart and muscle, consistent with global analyses of gene expression (Figures 1 – 3). In heart, unique CR responses of C57BL/6 mice included increased expression of genes associated with inactivity or denervation (Figure S5G) and decreased expression of genes connected to IGF-1 signaling (e.g., *Igfbp5*, *Igfbp4*, *Igf1*; Figure S5H). Loss of *Igf1* expression in C57BL/6 mice was likely an acute effect or immediate response to CR, since decreased *Igf1* can also be demonstrated from a prior microarray study (GSE68646) of cardiac tissue in young (10-12 week) C57BL/6 males subjected to only 1 week of 30% CR (Figure S6) [50]. Top-ranked increased genes in C57BL/6 heart were *Cyfip2*, *Ptgds* and *Scn5a*, while top-ranked decreased genes were *Bmp10*, *Ucp1* and *Myl7* (Figures S7A – S7D). In muscle, C57BL/6-unique responses to CR included increased expression of genes related to development (Figure S5I) and decreased expression of genes with metabolic functions (Figure S5J). The top-ranked increased genes in muscle were *Nnat*, *Lep* and *Tnni1*, and similarly the top-ranked decreased genes were *Otub2*, *Aldh1a2* and *Ndufb2* (Figures S7E – S7H)).

### Shared and strain-specific CR responses among genes belonging to longevity-regulating pathways

**Figure 4:**
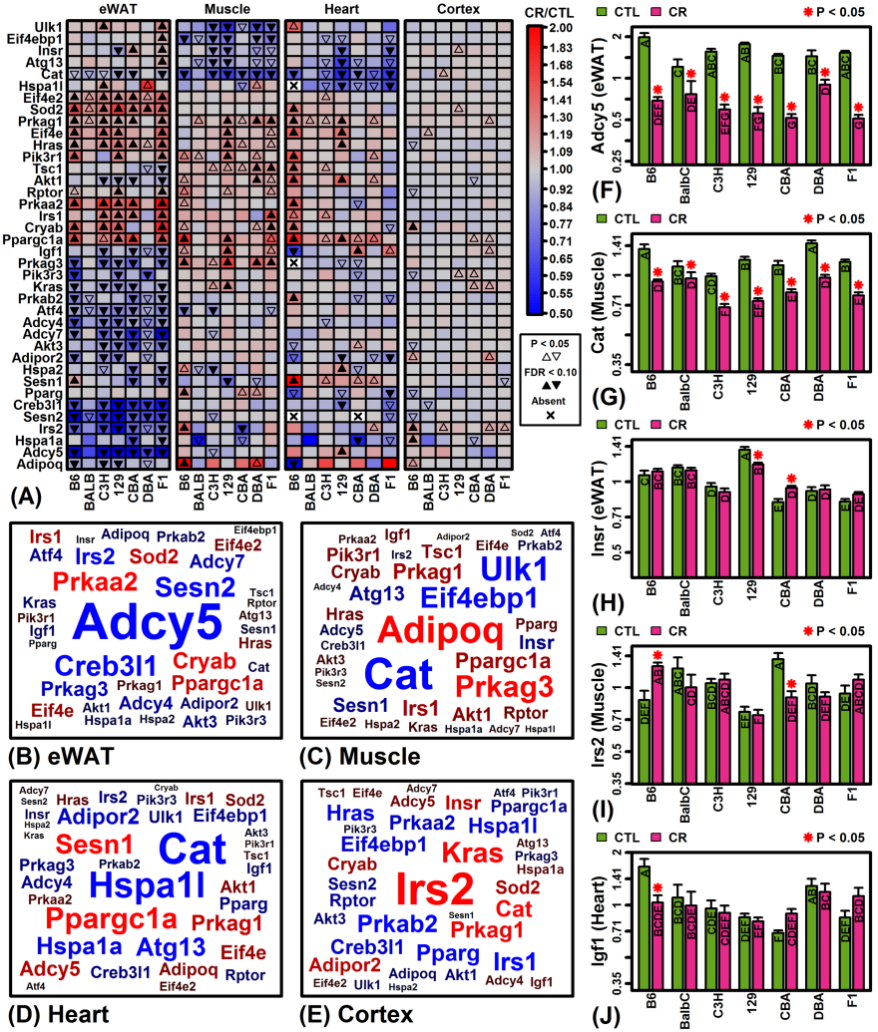
**KEGG longevity-regulating pathways (hsa04211 and hsa04213).** (**A**) Selected longevity pathway genes. The top 38 pathway genes were chosen to include those most frequently altered by CR across mouse strains and tissues. (**B – E**) Gene clouds. The size of gene symbols is proportional the median FC (CT/CTL) observed among strains for the indicated tissue (red: up-regulated; blue: down-regulated). Gene sizes are scaled separately for each tissue and thus not comparable across tissues. (**F**) adenylate cyclase 5 (*Adcy5*). (**G**) catalase (*Cat*). (**H**) insulin receptor (*Insr*). (**I**) insulin receptor substrate 2 (*Irs2*). (**J**) insulin-like growth factor 1 (*Igf1*). In (F) – (J), asterisks indicate that the CR treatment differs significantly from the CTL treatment for a given strain (P < 0.05). Treatments that share the same letter do not differ significantly (P < 0.05, Fisher’s least significant difference).

Pathways mediating longevity responses to CR include those linked to insulin-like growth factor I (IGF-1), sirtuins, and/or target of rapamycin (TOR) [51]. The KEGG longevity-regulating pathways provide one conceptual model for how such pathways interact and defines connections between genes and pathway components (KEGG identifiers hsa04211 and hsa04213) [52]. We identified genes associated with the KEGG longevity-regulating pathways most frequently altered by CR across the 7 strains and 4 tissues (Figure 4). Genes associated with these pathways were often consistently altered across strains, particularly with respect to eWAT (e.g., *Adcy5*, *Cat*, *Sesn2*, *Sod2*, *Eif4e2*; Figure 4A and 4B). Expression of catalase (*Cat*) was also consistently down-regulated by CR across strains in muscle and heart (Figures 4A, 4C, 4D and 4G). On the other hand, there were many instances in which longevity-regulating genes were altered in some strains but not others (Figure 4A). Genes with divergent response patterns among strains were in some cases linked to the insulin/IGF-1 system, such as *Insr* (eWAT), *Irs2* (muscle) and *Igf1* (heart) (Figures 4H – 4J).

### Olfactory receptor gene expression is uniquely elevated by CR in F1 heart and C3H muscle

**Figure 5:**
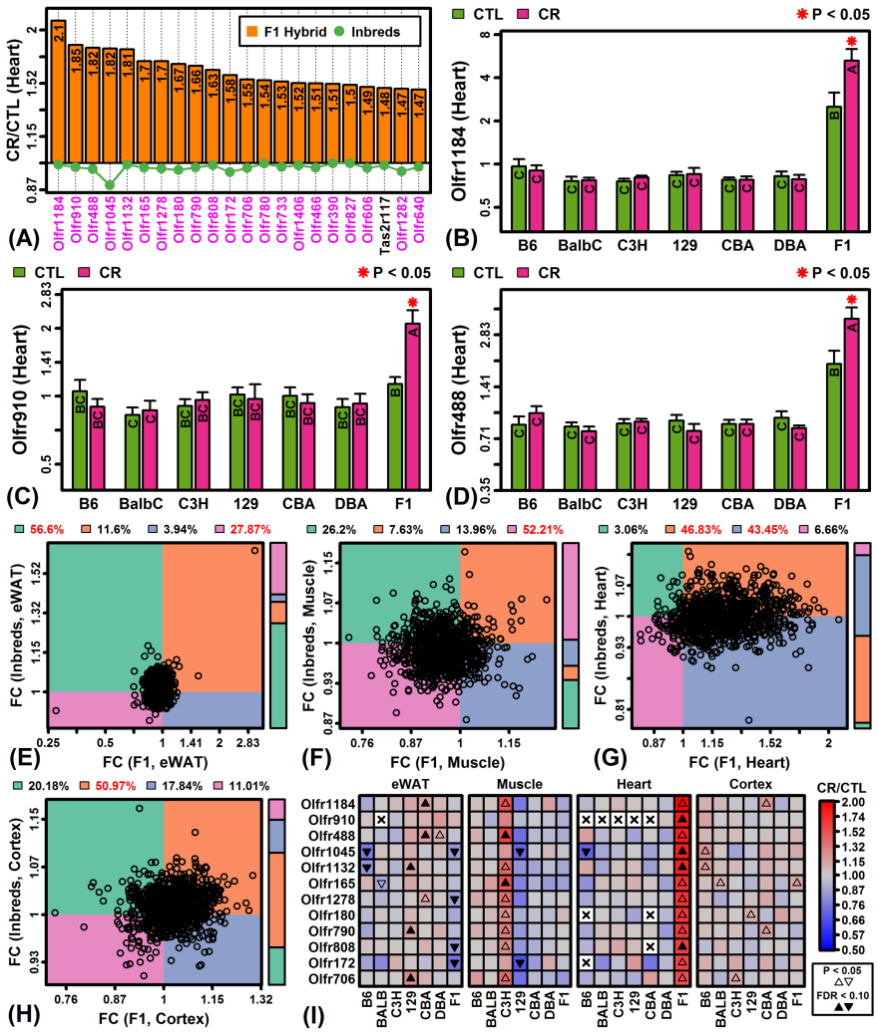
**CR specifically increases expression of olfactory receptor genes in F1 mouse heart tissue.** (**A**) Top-ranked genes most specifically increased by CR in heart tissue of F1 mice. Olfactory receptor genes are shown in magenta font (lower margin). The listed genes were significantly elevated in F1 heart tissue (FDR < 0.10; FC > 1.50) with significant strain-by-diet interaction effect (F1 mice vs. other strains; FDR < 0.15). The green line denotes the average FC of the 6 inbred strains. (**B, C, D**) Olfactory receptors 1184, 910 and 488 (*Olfr1184*, *Olfr910*, *Olfr488*). Asterisks indicate that the CR treatment differs significantly from the CTL treatment for a given strain (P < 0.05). Treatments that share the same letter do not differ significantly (P < 0.05, Fisher’s least significant difference). (**E, F, G, H**) Olfactory receptor FC scatterplot comparison (F1 vs. inbred mice). Each point represents FC estimates for one olfactory receptor gene (horizontal axis: F1 mice; vertical axis: average FC of inbred mice). The color bar (right) indicates the proportion of genes within each quadrant. The percentage of genes in each quadrant is indicated in the top margin (red: percentage significantly greater than 25%, P < 0.05, Chi-square test). (**I**) Top-ranked 12 olfactory receptor genes specifically expressed in F1 heart tissue.

Global analysis of CR responses provided evidence for a group of genes uniquely up-regulated by CR in C3H muscle and F1 heart (Figures 1G and 3A). These strain/tissue combinations also exhibited the second-largest number of genes with strain-by-diet interaction effects (Figures S5B and S5C). Closer inspection revealed that nearly all genes most specifically up-regulated by CR in these cases encoded olfactory receptors. In F1 heart, for example, 21 of the 22 genes most specifically up-regulated by CR encoded olfactory receptors (Figure 5A). Those genes demonstrating the strongest patterns, such as *Olfr1184*, *Olfr910* and *Olfr488*, were uniquely elevated by CR in F1 heart but were also expressed at higher levels in F1 mice regardless of diet (Figures 5B – 5D). Overall, 90% of genes encoding olfactory receptors were elevated by CR in F1 heart tissue with most genes not similarly altered in other strains (Figure 5G). This pattern was unique to F1 heart tissue and not observed in eWAT, muscle or cortex (Figures 5E, 5F and 5H). The set of olfactory receptor genes elevated in F1 heart were similarly elevated in C3H muscle but the same trends were not observed in other strain/tissue combinations (Figure 5I).

### Transcriptional responses to CR in mouse eWAT are negatively correlated with those in human scWAT

The laboratory mouse is a cornerstone for experimental aging research although humans and mice may differ in their CR diet responses [53]. In humans, there is no WAT depot that is strictly analogous to mouse eWAT [54], but several studies have evaluated gene expression responses to CR in scWAT [38–46]. We therefore compared CR responses in eWAT from mice to those observed for orthologous genes in scWAT samples from 28 experiments in which human subjects followed a CR diet (Table S2).

**Figure 6:**
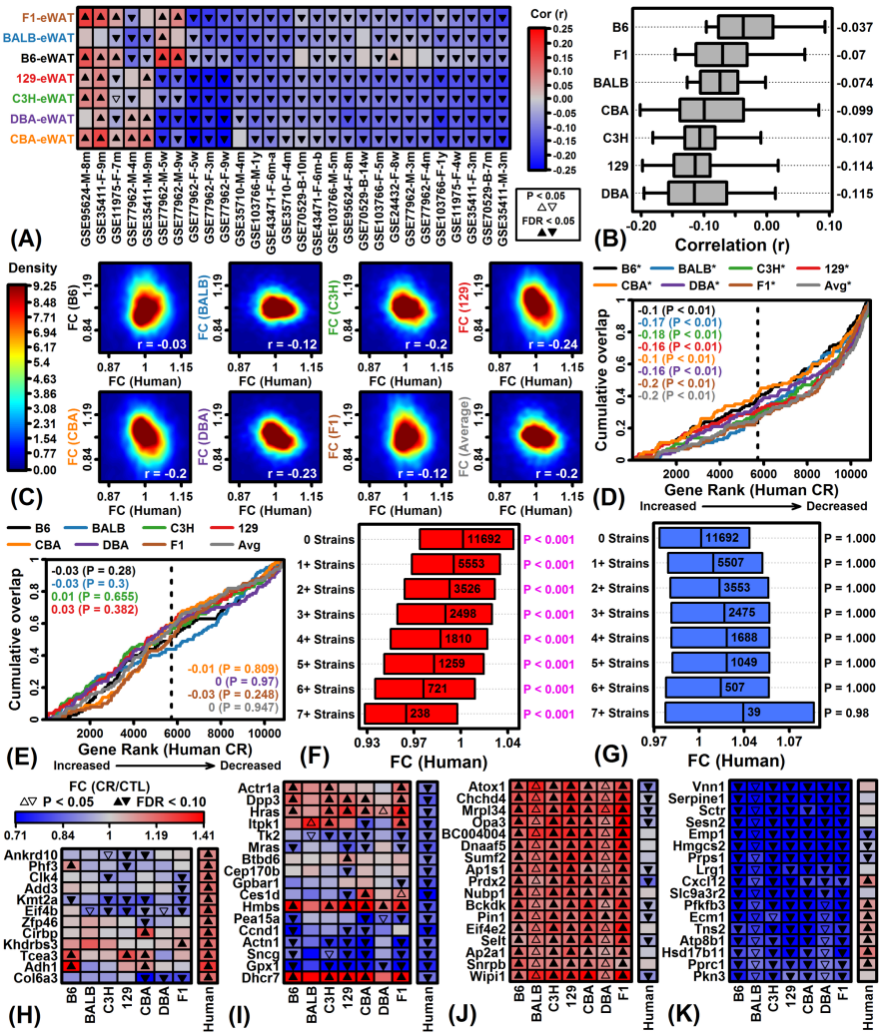
**Comparison of CR responses in mouse eWAT and human scWAT.** (**A**) Spearman rank correlations. FC estimates (CR/CTL) in 7 mouse strains (left margin) were compared to those in 28 human experiments (bottom margin). Bottom labels indicate GEO accession identifier, sex, and length of dietary intervention. (**B**) Correlation estimates by strain. Bars span the middle 50% of correlations for each strain (whiskers: middle 80%; right margin: median correlation). (**C**) FC scatterplots. FC estimates in each strain are compared to average FC estimates across the 28 human experiments. Colors denote gene density (see scale; lower right: Spearman rank correlation). (**D**) Gene set enrichment analysis (GSEA) of top 100 CR-increased genes in each mouse strain. (**E**) GSEA of top 100 CR-decreased genes in each mouse strain. In (D) and (E), genes were ranked according to their expression change with CR in humans (horizontal axis) and cumulative overlap was examined with respect to 100 CR-increased/decreased genes from each strain (vertical axis) (*P < 0.05, upper margin labels; enrichment statistics with p-values listed in each figure). Positive enrichment statistics indicate significant overlap with respect to genes increased by CR in human scWAT, while negative statistics indicate significant overlap with respect to genes decreased by CR in human scWAT (dashed vertical line: number of CR-increased genes in human, FC > 1.00). (**F**) Human FC estimates of genes increased by CR in multiple mouse strains. (**G**) Human FC estimates of genes decreased by CR in multiple mouse strains. In (F) and (G), bars span the middle 50% of human FC estimates. Significant p-values indicate that the median human FC estimate is significantly different from 1.00 (Wilcoxon rank sum test). (**H, I**) Genes most strongly altered by CR in human scWAT. (**J, K**) Genes most consistently altered by CR across 7 mouse strains (eWAT).

In 25 of 28 experiments, gene expression responses in most or all mouse strains were negatively correlated with human CR responses (Figure 6A). The average correlation across 28 experiments was negative for each strain (-0.115 ≤ *r_s_* ≤ -0.037) but was least negative with respect to C57BL/6 mice (*r_s_* = -0.037) (Figure 6B). Consistent with this, CR responses in each strain were negatively correlated with the average meta-response observed across the 28 human experiments (Figure 6C). The 100 genes most strongly increased by CR in each mouse strain were significantly enriched with respect to CR-decreased genes from humans (Figure 6D; P < 0.01 for all strains), although there were no significant trends with respect to the 100 genes most strongly decreased by CR in each mouse strain (Figure 6E; P ≥ 0.248). Interestingly, genes increased by CR in eWAT from multiple mouse strains were more strongly decreased by CR in human scWAT (Figure 6F). Genes decreased by CR in all 7 mouse strains were conversely increased by CR in humans, although this trend was non-significant (Figure 6G). Among genes most strongly altered by CR in humans, some exhibited consistent trends in mouse (*Tce3*, *Sncg*, *Gpx1*) but others were oppositely regulated by CR (*Kmt2a*, *Hmbs*, *Dhcr7*) (Figures 6H and 6I). None of the genes most consistently increased by CR across strains were similarly altered in humans (e.g., *Atox1*, *Chchd4*, *Mrpl34*; Figure 6J), and likewise, most genes consistently decreased by CR across strains were not similarly altered in humans (e.g., *Pfkfb3*, *Ecm1*, *Tns2*; Figure 6K).

**Figure 7:**
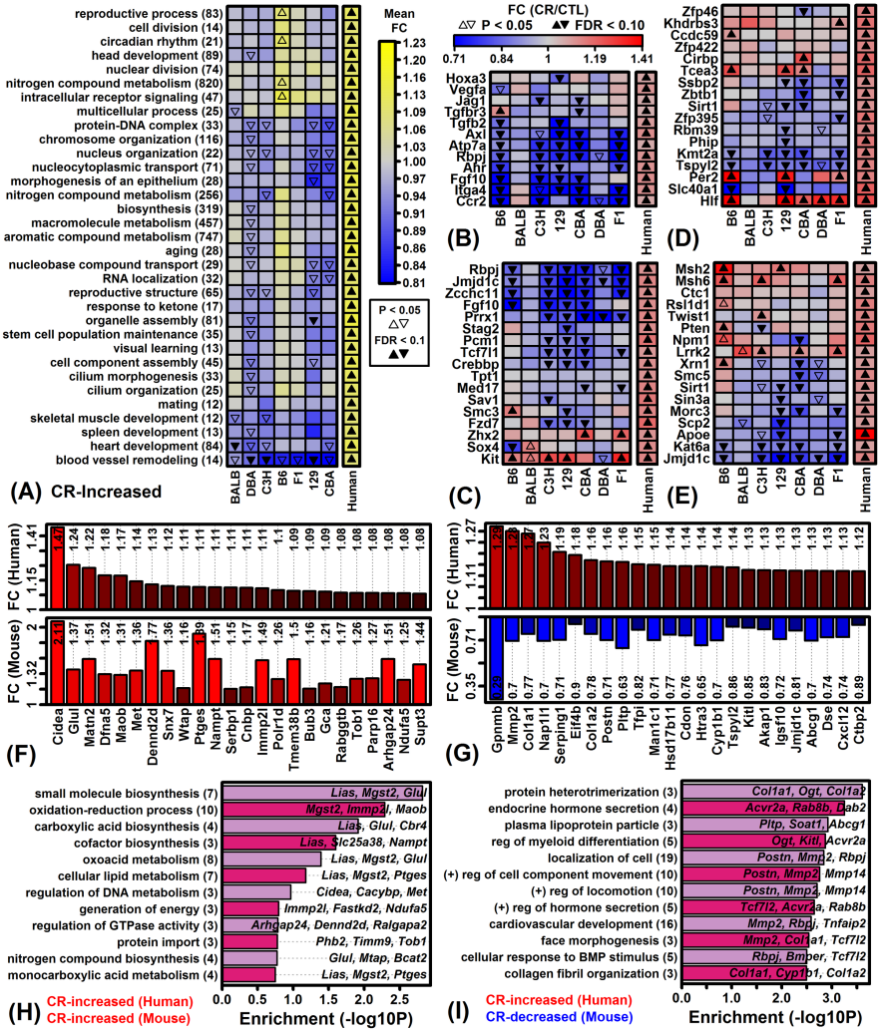
**Genes increased by CR in humans and Gene Ontology-based mouse comparison.** (**A**) GO BP terms most strongly enriched among genes increased by CR across 28 human experiments. (**B**) Genes associated with blood vessel remodeling (GO:0001974). (**C**) Genes associated with stem cell population maintenance (GO:0019827). (**D**) Genes associated with biosynthetic process regulation (GO:0009889). (**E**) Genes associated with aging (GO:0007568). (**F**) Genes most strongly increased by CR in humans and mice. (**G**) Genes increased by CR in humans but decreased in mice. In (F) and (G), color-coded bars show average FC estimates in humans (top) and mice (bottom). Average FC estimates are listed within each figure. (**H**) GO BP terms enriched among 70 genes increased by CR in humans and mice. Genes were increased by 5% on average in humans (FDR < 0.10) and increased by CR with respect to at least 6 of 7 mouse strains (P < 0.05 per strain). (**I**) GO BP terms enriched among 115 genes increased by CR in humans but decreased by CR in mice. Genes were increased by 5% on average in humans (FDR < 0.10) and decreased by CR with respect to at least 6 of 7 mouse strains (P < 0.05 per strain). In (H) and (I), the number of genes associated with each GO BP term is listed in parentheses (left margin) and example genes are listed within the figure.

To provide finer-scale comparison of mouse and human CR responses, correspondence was evaluated with respect to Gene Ontology (GO) Biological Process (BP) terms most strongly enriched among genes robustly elevated by CR in human scWAT (Figure 7A). This identified processes for which associated genes were increased by CR in humans and decreased by CR in most mouse strains, e.g., blood vessel remodeling (Figure 7B), stem cell population maintenance (Figure 7C), biosynthetic process regulation (Figure 7D) and aging (Figure 7E). Among 70 genes increased by CR in humans and mouse strains (Figure 7F), enriched GO BP terms included oxidation-reduction process and biosynthesis of small molecules, carboxylic acid and cofactors (Figure 7H). Likewise, among 115 genes increased by CR in humans but decreased in mouse strains (Figure 7G), enriched GO BP terms included protein heterotrimerization, endocrine hormone secretion and plasma lipoprotein particle (Figure 7I).

**Figure 8:**
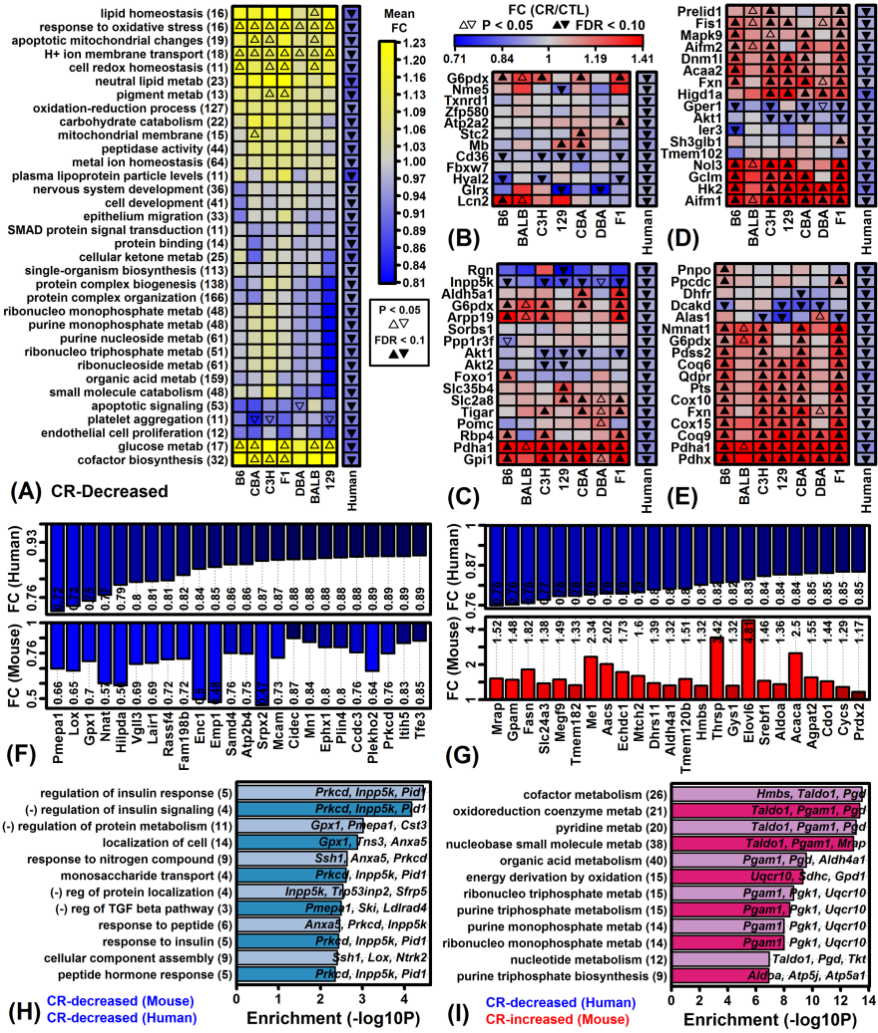
**Genes decreased by CR in humans and Gene Ontology-based mouse comparison.** (**A**) GO BP terms most strongly enriched among genes decreased by CR across 28 human experiments. (**B**) Genes associated with response to oxidative stress (GO:0006979). (**C**) Genes associated with glucose metabolic process (GO:0006006). (**D**) Genes associated with apoptotic mitochondrial changes (GO:0008637). (**E**) Genes associated with cofactor biosynthesis (GO:0051188). (**F**) Genes most strongly decreased by CR in humans and mice. (**G**) Genes decreased by CR in humans but increased in mice. In (F) and (G), color-coded bars show average FC estimates in humans (top) and mice (bottom). Average FC estimates are listed within each figure. (**H**) GO BP terms enriched among 74 genes decreased by CR in humans and mice. Genes were decreased by 5% on average in humans (FDR < 0.10) and decreased by CR with respect to at least 6 of 7 mouse strains (P < 0.05 per strain). (**I**) GO BP terms enriched among 233 genes decreased by CR in humans but increased by CR in mice. Genes were decreased by 5% on average in humans (FDR < 0.10) and increased by CR with respect to at least 6 of 7 mouse strains (P < 0.05 per strain). In (H) and (I), the number of genes associated with each GO BP term is listed in parentheses (left margin) and example genes are listed within the figure.

GO BP terms most strongly associated with genes decreased by CR in humans were identified and a term-by-term comparison was made to mouse CR responses (Figure 8A). Genes associated with platelet aggregation were down-regulated by CR with respect to both humans and mice (Figure 8A). However, contrasting patterns were observed with respect to genes associated with response to oxidative stress (Figure 8B), glucose metabolic process (Figure 8C), apoptotic mitochondrial changes (Figure 8D), and cofactor biosynthesis (Figure 8E). Among 74 genes decreased by CR in both humans and mice, there was significant enrichment with respect to GO BP terms linked to regulation of insulin response, protein metabolism and monosaccharide transport (Figure 8H). Likewise, among 233 genes decreased by CR in humans but increased in mice, we noted significant enrichment with respect to energy derivation by oxidation and metabolism of cofactors, oxidoreduction coenzymes, pyridine, nucleobase small molecules, organic acids, ribonucleoside triphosphate, purine triphosphate and purine monophosphate (Figure 8I).

### Effects of CR on the expression of genes related to stem cell maintenance, blood vessel remodeling and lipid metabolism in human scWAT are not replicated in 4 mouse WAT depots

We noted an inverse association between CR responses in mouse eWAT and human scWAT (Figure 6). These are the most commonly studied forms of WAT in mice and humans, respectively, although potentially CR responses in other mouse depots would correspond better to those in human scWAT [54]. To evaluate this possibility, we compared CR responses in human scWAT and 4 WAT depots from obese C57BL/6 male mice (eWAT, scWAT, perirenal [prWAT], mesenteric [mesWAT]). In these experiments, samples were obtained from mice fed a high fat diet with 25% CR enforced for 1 – 60 days (see GEO series accession GSE30534). Applying a strict FDR threshold of 0.10, we did not identify any significant CR-regulated genes. However, given a weaker threshold of P < 0.05 (with FC > 1.50 or FC < 0.67), we identified between 4 and 403 genes with CR-regulated expression, depending upon the WAT depot examined and the CR duration (Figures S8 and S9).

Consistent with the above findings, CR responses in C57BL/6 eWAT were negatively correlated with those in human scWAT (Figure S10A and S10B). We expected improved correspondence when comparing scWAT responses in both species, but the average correlation was still negative on average across the 28 human experiments (*r*_s_ = -0.016 for the 60 day CR response; Figure S10B). Moreover, genes associated with stem cell maintenance and blood vessel remodeling, although increased by CR in human scWAT (FDR < 0.10), were not correspondingly altered in mouse scWAT (P > 0.05 for all genes; Figures S11A, S11B and S11E). Likewise, genes related to hydrogen ion membrane transport and neutral lipid metabolism decreased by CR in human scWAT (FDR < 0.10) were not correspondingly altered in mouse scWAT (P > 0.05 for all genes; Figures S11C, S11D and S11F). Overall, CR responses in mouse prWAT best correlated with those in human scWAT, with positive correlations on average calculated for 5 of the 7 time points evaluated (Figure S10B). Consistent with this, the 100 genes most strongly increased by CR in C57BL/6J prWAT overlapped significantly with genes increased by CR in human scWAT (Figures S10C and S10D). Conversely, the 100 genes most strongly decreased by CR in C57BL/6J prWAT overlapped significantly with genes decreased by CR in human scWAT (Figures S10E and S10F).

## DISCUSSION

Mouse CR studies have frequently been limited to one strain (e.g., C57BL/6) and it has often been unclear whether conclusions should be generalized to other strains or translated to humans [13,14,17]. This study analyzed publically available microarray data (GSE75574) to compare short-term (14 week) CR responses in males from 7 mouse strains to distinguish shared and strain-specific CR responses in 4 tissues. The largest number of strain-specific CR responses was identified with respect to the C57BL/6 strain, indicating that responses in this strain may not be replicated in other genotypes. Such strain-specific responses can contribute to discrepant findings among laboratories, diminishing the apparent repeatability of preclinical research [13–15]. Our findings demonstrate this possibility and we expect that strain-specific effects identified here can inform the selection of background strain for studies targeting WAT, heart, muscle or neocortex [17]. To facilitate translation of mouse findings, we attempted to identify a mouse strain for which CR responses in eWAT best matched those of human scWAT, but unexpectedly responses in all strains were negatively correlated with those from human experiments (Figures 6A and 6B). This surprising outcome may be explained by differences in the WAT depots examined for each species [54,55], or alternatively may reflect genuine species differences related to WAT metabolism in response to caloric deficit [56,57]. In either case, our findings raise the concern that the most commonly studied WAT depots from mice (epididymal) and humans (subcutaneous) are poorly analogous. This has implications regarding the interpretation and design of studies that aim to understand WAT responses to dietary interventions in mice.

A small number of inbred strains have been disproportionately utilized in biomedical research [19], but laboratory mice harbor considerable genetic diversity such that the concurrent analysis of multiple strains provides an opportunity to better understand genetic factors shaping CR responses [8,9]. In our analysis, strain was consistently a stronger factor than diet in accounting for gene expression variation (Figures 1A and 1B), and from multivariate analyses we could identify strains with distinctive global CR response patterns (e.g., B6-heart, B6-muscle, F1-heart; Figure 3D). By integrating trends from thousands of genes, our findings can help guide the choice of background strain for experiments depending upon the organ of focus. In cardiac tissue from C57BL/6 mice, for example, we identified a larger number of strain-specific CR responses than any of the other 27 strain-tissue combinations (1805 genes with FDR < 0.10; Figure S5C). Among these, several longevity-associated genes were uniquely up-regulated by CR in C57BL/6 heart (e.g., *Prkab2*, *Cryab*, *Prkaa2*, *Sod2*) and other such genes were uniquely down-regulated (e.g., *Igf1*, *Igfbp4*, *Igfbp5*, *Adipoq*, *Akt3*) (Figures 4A, 4J and Figure S5H). Strain-specific down-regulation of *Igf1*, *Igfbp4* and *Igfb5* by CR in C57BL/6 mice suggests a unique pattern of cardiac IGF-1 pathway dysregulation differing from other strains. Local IGF-1 signaling in heart has anti-apoptotic and regenerative effects [58] and is associated with improvements in cell growth, contractility, cardiac output, stroke volume, ejection fraction, functional recovery following myocardial infarction and insulin sensitivity [59,60]. CR-driven declines in local cardiac *Igf1* mRNA levels in C57BL/6 mice may therefore have repressive effects on cardiac function absent in other mouse strains. Compared to C57BL/6, cardiac effects of CR on gene expression in Balbc/J, C3H/HeJ, 129S1/SvImJ and CBA/J mice were more mutually consistent (Figures 2C and 2E) and we identified no significant strain-specific effects at a stringent FDR threshold (Figure S5C). These strains may provide preferred backgrounds for studying cardiac-specific effects of CR and possibly other dietary interventions as well.

Olfactory receptors (ORs) comprise the largest vertebrate gene superfamily consisting of more than 900 human genes [61] and 1296 mouse genes [62]. These genes encode G-protein coupled receptors (GPCRs) that are mostly expressed on olfactory sensory neurons of the nasal epithelium. In recent decades, however, expression of ORs has been discovered in germ cells [63] and postnatal cardiac cells [64], and RNA sequencing studies have increasingly identified expression of ORs in other non-chemosensory tissues (“ectopically expressed ORs”) [65]. In this study, OR-encoding genes showed a strong trend towards strain-specific CR responses, with many increased by CR specifically in C3H muscle and B6C3F1/J heart (Figures 5G and 5I). It is unclear why OR expression is modulated by CR in such a strain-specific fashion, although previous studies have demonstrated decreased expression of ORs in CR-fed mice and in long-lived mice lacking the adenylate cyclase 5 gene (129/Sv background) [66]. The importance of ORs in aging was first suggested by invertebrate genetic studies, which demonstrated that OR loss-of-function mutations increase longevity in *Caenorhabditis elegans* [67] and *Drosophila melanogaster* [68]. In vertebrates, ectopically expressed ORs appear to have diverse functions including glucose homeostasis and metabolism [69,70]. An allele for OR14J1C, for example, has been associated with increased type 1 diabetes risk [71], and mice lacking the *Olfr1393* gene exhibit decreased Sglt1 function in proximal kidney tubules leading to decreased glucose reabsorption and glycosuria [72]. In cardiac tissue, OR51E1 acts as a receptor for medium-chain fatty acids, which stimulate OR51E1 to negatively regulate heart inotropy [70]. Through these and other mechanisms, ORs may mediate metabolic responses to CR, and given our findings we anticipate that these effects would be strain-specific and distinctive in C3H muscle and B6C3F1/J heart (Figure 5I).

The extrapolation of findings from mouse CR studies to humans has been controversial with some investigators arguing that CR responses in mice differ fundamentally from humans in important respects [31,32]. To address this issue, we focused on WAT to compare CR responses in eWAT from each mouse strain to human scWAT responses. Our findings do not suggest an absence of association, but rather demonstrate a significant *negative* correlation (Figures 6A and 6B). Moreover, genes most consistently up- or down-regulated by CR across strains were more likely to exhibit opposite responses in human scWAT (Figures 6F and 6G). We propose two interpretations for these findings that are not mutually exclusive. First, human studies evaluated periumbilical scWAT whereas CR responses in 7 strains were evaluated in perivisceral eWAT [54]. In humans, periumbilical scWAT is an accessible site that is cosmetically acceptable for obtaining WAT samples, such that periumbilical scWAT has been the most widely evaluated in clinical weight loss studies [38–46]. Similarly, in mice, eWAT is the largest and most widely studied depot, but may not be strictly analogous to any depot found in human [54]. Compared to other mouse depots, eWAT appears to have increased rates of glucose and lipid metabolism, adipocyte size and stress resistance protein abundance [73]. Effects of CR on eWAT may therefore be dissimilar to other depots, such that other WAT sampling sites in mouse would yield better agreement with human clinical data. There was limited support for this possibility, since our analysis of an independent dataset indicated that CR responses in human scWAT were better correlated with responses in C57BL/6 male scWAT and mesWAT (-0.073 ≤ *r*_s_ ≤ 0.064), although the best correlations were observed with respect to mouse prWAT (-0.013 ≤ *r*_s_ ≤ 0.091) (Figure S10B). These trends suggest that, at least for C57BL/6 males, correspondence between effects of CR on WAT from mice and humans is influenced by depot, although in all cases we observed only modest mouse-human correlations (*r*_s_ < 0.10).

A second interpretation of our findings is that WAT metabolism in humans and mice differs in biologically important ways under hypocaloric conditions. After all, rodents and humans differ in their grossly observable response to reduced calorie diets, with CR-fed rodents exhibiting a “scavenger response” characterized by increased spontaneously physical activity [74,75], whereas in humans this response is absent and in contrast CR reduces physical activity [76,77]. These divergent behavioral responses to CR may correspond to different short-term levels of energy expenditure, different metabolic demands, and thus different levels of fatty acid breakdown. Consistent with this, genes up-regulated by CR in human scWAT were associated with stem cell maintenance, angiogenesis, and cell division (Figure 7A), whereas down-regulated genes were linked to energy derivation via oxidation (Figure 8I). In mouse eWAT, scWAT, prWAT and mesWAT, CR had opposite or non-significant effects on the expression of orthologous genes associated with these processes (Figures 7A, 8I, Figure S11). These trends suggest a human CR response favoring adipose tissue maintenance, contrasting with a rodent response favoring WAT breakdown and fatty acid metabolism. This difference may be driven by lower energy expenditure with CR in humans, although in addition humans may rely more heavily on other energy sources apart from adipose, e.g., amino acids, gluconeogenesis, muscle glycogen stores, or ketone production. Interestingly, for example, CR in human scWAT increased expression of genes associated with “response to ketone”, whereas expression of orthologous genes were not altered by CR in eWAT from any mouse strain (Figure 7A). Ultimately, these mechanisms may allow humans to better maintain fat mass under hypocaloric conditions, providing a physiological reserve that can be drawn upon in the event of severe famine at a later date. This is an appealing explanation for our findings since it fits with the idea that humans harbor “thrifty” genes to ensure efficient energy utilization and maintenance of adipose reserve, which in modern settings has proven deleterious as contributing to obesity and obesity-related disease [56,57].

This study evaluated the most comprehensive gene expression dataset available for evaluating effects of CR in multiple mouse strains [37]. A limitation of our findings is that analyses were carried out with respect to male mice only. Interventions influencing mouse longevity and/or healthspan may have sex-specific effects, and thus our observations in males may not be applicable to females [78]. Indeed, some evidence indicates that CR may have stronger growth-inhibiting effects in males [79], whereas females maintain body weight better with CR and thus appear more energy efficient [80]. Secondly, the dataset we considered allowed us to evaluate effects of CR in young mice, with CR applied between 8 and 22 weeks of age. The effects of CR, however, may differ in young versus old mice [81], and in particular effects of CR at a young age may be unrevealing with regard to age-associated pathology. One consistent effect of CR emerging from gene expression meta-analyses, for example, has been inhibition of inflammatory gene expression patterns [47]. However, since inflammation-associated gene expression tends to increase at older ages in mice, repression of such patterns by CR may not be apparent in younger mice [47]. Along these lines, strain-specific CR responses related to longevity may be difficult to discern based upon our analysis. For example, CR appears to have an especially robust and positive effect on the lifespan of hybrid strains such as B6C3F1/J [20], whereas CR is known to have weak or even negative effects on the lifespan of DBA/2J mice [21–23]. Despite this, neither B6C3F1/J nor DBA/2J was a strong outlier in our analyses and comparatively few strain-specific expression responses were identified in these strains (Figures S5A – S5D). However, if effects of CR had been evaluated in older mice, following the onset of age-related morbidities, a larger number of strain-specific expression responses may have been observed in B6C3F1/J nor DBA/2J mice, potentially with more distinctive shifts in the expression of longevity-associated genes (e.g., *Cat*, *Prkag1*, *Irs1*, *Ppargc1a*; Figure 4A).

The proposed healthspan and lifespan benefits of CR observed for decades in mice and rats have so far been partially replicated in primate studies. In rhesus monkeys, effects of CR on survival have varied, with treatment comparisons (CR versus control) supporting a range of outcomes spanning from a 12% decrease to 24% increase in median survival for CR-fed monkeys (Kaplan–Meier estimates) [82]. In humans, effects of CR on survivorship are difficult to discern, but favorable shifts in biomarkers and physiological performance have been reported in non-obese trial participants adhering to a CR diet [83]. In this evolving context, laboratory mice will continue to play a valuable role as a flexible experimental model, offering diverse genetic tools and environmental controls that could never be achieved in human studies. Ultimately, however, appropriate use of this tool requires an understanding of strain effects and the biology underlying the mouse and human “translation gap” [84]. The current study provides new insights by characterizing strain-specific CR responses and evaluating whether such responses are replicated in human datasets. This facilitates an informed interpretation of mouse findings from a translational perspective and thus enhances the utility of mice as a tool for understanding dietary responses.

## MATERIALS AND METHODS

### Microarray analysis of gene expression responses to CR in 7 mouse strains

The complete dataset included 448 microarray samples available from the Gene Expression Omnibus (GEO) database under the series accession GSE75574 (7 strains × 4 tissues × 2 diets × 8 mice per treatment group = 448 samples total) [37]. A complete description of animal husbandry and dietary protocols has been reported previously [37]. In brief, male mice were purchased from the Jackson lab at 6 weeks of age and provided AIN93M rodent diet (84 kcal/week). Mice were randomized to either CR or *ad lib* diet treatments starting at 8 weeks of age with sacrifice and tissue collection at 22 weeks. For the Balbc/J, C3H/HeJ, CBA/J, DBA/2J and B6C3F1/J strains, CR-fed mice received a dietary allowance reduced in weight by 23 – 25% compared to CTL-fed mice (Table S1). For CR-fed C57BL/6J and 129S1/SvImJ mice, the dietary allowance was initially reduced by 25% (weeks 8 – 14) and then decreased further by 42% (weeks 14 – 22) to prompt weight loss similar to that in other strains (Table S1) [37]. Regarding body weights at week 22, effects of CR were similar with 16 – 24% reduction compared to CTL, although Balbc/J was an exception with average weight of CR-fed mice reduced by only 4% (Table S1). At the conclusion of dietary interventions (week 22), mice were fasted overnight prior to sacrifice by cervical dislocation [37]. The 4 tissues collected for RNA analysis were eWAT, skeletal muscle (gastrocnemius), heart and brain neocortex.

### Data processing and normalization

Gene expression profiling was performed using the high-density Affymetrix Mouse Gene 1.0 ST array platform for whole transcript expression analysis. Material from one biological sample was hybridized to a single microarray, yielding a total of 448 raw CEL files downloaded under the GSE75574 accession. Raw CEL files were normalized using the robust multichip average (RMA) algorithm for gene-level expression intensity summarization (R package: oligo; function: RMA) [85]. Expression estimates were calculated for 35556 probe identifiers, of which 20353 could be associated with a protein-coding gene (R annotation package: mogene10sttranscriptcluster.db). These 20353 probe identifiers were collectively associated with 18763 unique protein-coding genes. To limit redundancy in downstream analyses, a single probe identifier was chosen to represent each protein-coding gene [86]. For each tissue, the probe identifier with highest average expression across all samples was chosen as the representative in cases where multiple probe identifiers were associated with the same gene symbol. Following these steps, there remained 18763 probe identifiers, each of which was associated with a unique gene symbol.

### Microarray quality control

The initial set of 448 array samples was analyzed to identify outliers or poor-quality hybridizations. Microarray samples for each tissue were clustered and each sample was plotted with respect to the first two principal components calculated from genome-wide expression intensity estimates (Figures S12 – S15). Additionally, the probe-level model metrics normalized unscaled standard error (NUSE) and relative log expression (RLE) were calculated for each sample, with low-quality samples suggested by NUSE median estimates differing substantially from 1 or RLE median estimates differing from 0 (Figures S12 – S15) [87]. A total of 12 samples were removed because they were identified as outliers or probe-level metrics suggested poor-quality array hybridizations (excluded eWAT samples: GSM1959516, GSM1959551; excluded muscle samples: GSM1959292, GSM1959322, GSM1959354, GSM1959359; excluded heart samples: GSM1959406, GSM1959427, GSM1959457, GSM1959468; excluded cortex samples: GSM1959158, GSM1959224). Thus, the final dataset used for subsequent analyses included 436 microarray samples among the 4 tissues (448 – 12 = 436 samples).

### Differential expression analyses

Differential expression analyses were performed to evaluate the effect of diet (CR versus *ad lib*) with respect to each strain and tissue (7 strains × 4 tissues = 28 strain-tissue combinations total). For a given sample, a gene was determined to have detectable expression if at least 50% of probe sets associated with that gene were expressed above-background at a significance level of P < 0.05, based upon a signed-rank test comparison between perfect match and mismatch probe intensities [88]. Differential expression testing was performed using only genes with detectable expression in at least 33% of the microarray samples available for a given CR versus *ad lib* comparison. Among the 28 strain-tissue combinations, this yielded between 14083 and 17883 protein-coding genes for inclusion in differential expression analyses (average of 16474 genes). A total of 15490, 15375, 13588 and 17406 genes met criteria for all comparisons with respect to eWAT, muscle, heart and cortex, respectively. Likewise, 13129 protein-coding genes were consistently expressed in samples from each tissue type and included in analyses in each of the 28 strain-tissue combinations. Differential expression between CR and *ad lib* samples was evaluated using empirical Bayes linear models and moderated t statistics as implemented in the R limma package [89]. To control the false discovery rate for each CR versus *ad lib* comparison, raw p-values were adjusted using the Benjamini-Hochberg method [90]. Unless otherwise indicated, genes were considered to be differentially expressed if the FDR was less than 0.10 and estimated fold-change (FC) was greater than 1.50 or less than 0.67. To evaluate the accuracy of our analyses, we compared microarray FC estimates to those previously reported [37] and obtained using RT-PCR for 11, 10 and 7 genes in eWAT, muscle and heart, respectively (C57BL/6J strain). As expected, microarray-based FC estimates were less extreme than those obtained by RT-PCR, likely reflecting reduced dynamic range of microarrays compared to PCR assays [91]. However, for each tissue examined, we noted a strong positive correlation between FC estimates obtained by microarray and RT-PCR (r ≥ 0.86; Figure S16).

### Strain-by-diet interaction effects

Linear models with moderated t statistics were also used to identify genes with strain-dependent CR responses (i.e., strain-by-diet interaction effects). For this approach, gene expression was modeled as a function of diet, strain and an interaction term (i.e., Expression = Diet + Strain + Diet*Strain) with analyses replicated for each of the 28 strain-tissue combinations. Diet was defined as a variable with value 1 for CR samples and 0 for *ad lib* samples. Likewise, strain was defined as a dummy variable with value 1 assigned to samples associated with a given strain and value 0 assigned to samples associated with any of the other 6 strains. The strain-by-diet interaction effect was then evaluated based upon p-values associated with coefficients estimated for the strain-by-diet model term (Diet*Strain). Interaction effects were analyzed only for genes with detectable expression in 33% of all microarray samples for each tissue (eWAT: 16398 genes; Muscle: 16634; Heart: 15416; Cortex: 17793).

### Meta-analysis of human scWAT responses to CR

We identified 28 experiments in which microarrays were used to analyze gene expression in periumbilical scWAT from human subjects before and after CR diet interventions (Table S2) [38–46]. In this context, an “experiment” is defined as two paired sets of microarray samples, including a baseline set obtained from scWAT of subjects prior to intervention and a post-intervention set obtained from subjects post-intervention. Following this definition, multiple experiments could be derived from the same study if repeated measures were obtained from subjects followed longitudinally over time (see below).

The 28 experiments include only dietary interventions with some protocols incorporating an additional exercise component, although we excluded any experiment in which a gastric bypass procedure was performed (Table S2). All dietary interventions were designed to induce weight loss with some experiments utilizing a baseline- and subject-specific calorie reduction protocol, and others utilizing a fixed protocol for all subjects with total calorie count ranging from 450 to 2000 kCal per day. Interventions ranged in duration from 4 weeks to 1 year with 10 of 28 experiments performed using males and 15 of 28 experiments performed using females (the sex was unknown for 3 experiments). Experiments varied in size from 3 to 40 subjects (average 10.9) with a total of 503 microarrays analyzed among all 28 experiments. For each experiment, we excluded from analyses the 15% of genes with lowest expression on average among all samples. For remaining genes and each patient, the difference in log2-transformed expression before and after treatment was calculated. For each gene, the average and standard error of these differences was calculated, and the experiment-specific significance of that difference was evaluated using linear models with moderated t-statistics [89].

A random effects meta-analysis model was applied to integrate results across the 28 experiments and to calculate a meta-signature for the effects of CR in human scWAT [92]. In some cases, 2 or more experiments had been obtained from repeated measures experiments and shared the same baseline set of samples. Results from such experiments cannot be regarded as independent. To obtain a filtered set of independent experiments, the 28 experiments were ranked according to the average standard error calculated among all genes with respect to the before versus after comparison. We filtered out any experiments sharing the same set of baseline samples, preferentially retaining those experiments with the lowest average standard error. This yielded a filtered set of 18 independent experiments with expression measurements available for 16504 genes. We excluded 3723 genes not measured in 6 or more of the 18 independent experiments, yielding a final set of 12781 genes for inclusion in the random effects meta-analysis model (R package: meta, R function: metagen). For this approach, the DerSimonian-Laird estimate was used to calculate a meta-FC for each gene with inverse variance weighting (i.e., greater weight assigned to experiments associated with a lower standard error estimate) [92]. To control for multiple hypothesis testing among the 12781 genes, p-values derived from the meta-analysis model were adjusted using the Benjamini-Hochberg method [90].

### Microarray analysis of multiple WAT depots in *ad lib* and CR-fed obese C57BL/6 mice

Our main focus was to compare effects of CR in mouse eWAT and human scWAT, since these were the most well studied in terms of CR gene expression responses in each species, respectively [54]. However, to replicate this comparison using multiple WAT depots from a given strain, gene expression was evaluated in 4 WAT depots (eWAT, scWAT, perirenal WAT [prWAT]; mesenteric WAT [mesWAT]) from obese C57BL/6 male mice provided a high fat diet and subjected to CR for 0, 1, 3, 7, 14, 21, 42 and 60 days (GEO series accession GSE30534).

The initial dataset included 312 samples with genome-wide expression evaluated using the Affymetrix Mouse Genome 430 2.0 *in situ* oligonucleotide array platform. The 312 samples were normalized using robust multichip average yielding expression intensities for 45101 probe sets [85]. Samples from each WAT depot were clustered and visualized with respect to principal component axes to identify outliers, and were additionally evaluated using quality control metrics to identify low-quality hybridizations (e.g., NUSE/RLE median/IQR, average background, Affymetrix scale factors) (Figure S17) [87]. A total of 6 samples were identified as outliers and/or poor quality hybridizations and removed from analyses (GSM757584, GSM757585, GSM757599, GSM757392, GSM757502 and GSM757465), leaving a total of 306 samples used in further analyses.

The 45101 probe sets were collectively associated with 20736 human genes. If multiple probe sets were associated with the same gene, a single probe set was chosen to represent the gene by selecting the probe set with highest average expression among all samples [86]. We therefore considered a total of 20736 probe sets, with each probe set representing a unique human gene. Differential expression analyses were performed for 28 comparisons (4 WAT depots × 7 time points = 28 CR vs. *ad lib* comparisons). For a given comparison, only genes with detectable expression in at least 33% of samples were evaluated, yielding between 14711 and 15939 human genes among the 28 comparisons. A signed-rank test comparison between perfect match and mismatch probe intensities was used to determine if expression intensities for each probe set were detectable above background levels [88]. Differential expression analyses were carried out using linear models and moderated t-statistics [89], with raw p-values adjusted using the Benjamini-Hochberg method [90].

## Supplementary Material

Supplementary File

## References

[1] Fontana L, Partridge L. Promoting health and longevity through diet: from model organisms to humans. Cell. 2015; 161:106–18. 10.1016/j.cell.2015.02.020 25815989 PMC4547605

[2] Osborne TB, Mendel LB, Ferry EL. The effect of retardation of growth upon the breeding period and duration of life of rats. Science. 1917; 45:294–95. 10.1126/science.45.1160.294 17760202

[3] McCay CM, Crowell MF, Maynard LA. The effect of retarded growth upon the length of life span and upon the ultimate body size. 1935. Nutrition. 1989; 5:155–71. 2520283

[4] Tuchweber B, Salas M. Experimental pathology of aging. Methods Achiev Exp Pathol. 1975; 7:167–226. 1105062

[5] Ingram DK, de Cabo R. Calorie restriction in rodents: caveats to consider. Ageing Res Rev. 2017; 39:15–28. 10.1016/j.arr.2017.05.008 28610949 PMC5565679

[6] Forster MJ, Morris P, Sohal RS. Genotype and age influence the effect of caloric intake on mortality in mice. FASEB J. 2003; 17:690–92. 10.1096/fj.02-0533fje 12586746 PMC2839882

[7] Harper JM, Leathers CW, Austad SN. Does caloric restriction extend life in wild mice? Aging Cell. 2006; 5:441–49. 10.1111/j.1474-9726.2006.00236.x 17054664 PMC2923404

[8] Liao CY, Rikke BA, Johnson TE, Diaz V, Nelson JF. Genetic variation in the murine lifespan response to dietary restriction: from life extension to life shortening. Aging Cell. 2010; 9:92–95. 10.1111/j.1474-9726.2009.00533.x 19878144 PMC3476836

[9] Petkov PM, Ding Y, Cassell MA, Zhang W, Wagner G, Sargent EE, Asquith S, Crew V, Johnson KA, Robinson P, Scott VE, Wiles MV. An efficient SNP system for mouse genome scanning and elucidating strain relationships. Genome Res. 2004; 14:1806–11. 10.1101/gr.2825804 15342563 PMC515327

[10] Sundberg JP, Berndt A, Sundberg BA, Silva KA, Kennedy V, Bronson R, Yuan R, Paigen B, Harrison D, Schofield PN. The mouse as a model for understanding chronic diseases of aging: the histopathologic basis of aging in inbred mice. Pathobiol Aging Age Relat Dis. 2011; 1:1. 10.3402/pba.v1i0.7179 22953031 PMC3417678

[11] Svenson KL, Von Smith R, Magnani PA, Suetin HR, Paigen B, Naggert JK, Li R, Churchill GA, Peters LL. Multiple trait measurements in 43 inbred mouse strains capture the phenotypic diversity characteristic of human populations. J Appl Physiol (1985). 2007; 102:2369–78. 10.1152/japplphysiol.01077.2006 17317875

[12] Yuan R, Tsaih SW, Petkova SB, Marin de Evsikova C, Xing S, Marion MA, Bogue MA, Mills KD, Peters LL, Bult CJ, Rosen CJ, Sundberg JP, Harrison DE, et al. Aging in inbred strains of mice: study design and interim report on median lifespans and circulating IGF1 levels. Aging Cell. 2009; 8:277–87. 10.1111/j.1474-9726.2009.00478.x 19627267 PMC2768517

[13] Churchill GA. Misleading results: don’t blame the mice. Science. 2014; 343:370. 10.1126/science.343.6169.370-a 24458625

[14] Lucanic M, Plummer WT, Chen E, Harke J, Foulger AC, Onken B, Coleman-Hulbert AL, Dumas KJ, Guo S, Johnson E, Bhaumik D, Xue J, Crist AB, et al. Impact of genetic background and experimental reproducibility on identifying chemical compounds with robust longevity effects. Nat Commun. 2017; 8:14256. 10.1038/ncomms14256 28220799 PMC5321775

[15] Collins FS, Tabak LA. Policy: NIH plans to enhance reproducibility. Nature. 2014; 505:612–13. 10.1038/505612a 24482835 PMC4058759

[16] Ioannidis JP. Why most published research findings are false. PLoS Med. 2005; 2:e124. 10.1371/journal.pmed.0020124 16060722 PMC1182327

[17] Sittig LJ, Carbonetto P, Engel KA, Krauss KS, Barrios-Camacho CM, Palmer AA. Genetic background limits generalizability of genotype-phenotype relationships. Neuron. 2016; 91:1253–59. 10.1016/j.neuron.2016.08.013 27618673 PMC5033712

[18] Ehret T, Torelli F, Klotz C, Pedersen AB, Seeber F. translational rodent models for research on parasitic Protozoa-a review of confounders and possibilities. Front Cell Infect Microbiol. 2017; 7:238. 10.3389/fcimb.2017.00238 28638807 PMC5461347

[19] Miller RA. Not your father’s, or mother’s, rodent: moving beyond B6. Neuron. 2016; 91:1185–86. 10.1016/j.neuron.2016.09.009 27657444 PMC5495110

[20] Swindell WR. Dietary restriction in rats and mice: a meta-analysis and review of the evidence for genotype-dependent effects on lifespan. Ageing Res Rev. 2012; 11:254–70. 10.1016/j.arr.2011.12.006 22210149 PMC3299887

[21] Hempenstall S, Picchio L, Mitchell SE, Speakman JR, Selman C. The impact of acute caloric restriction on the metabolic phenotype in male C57BL/6 and DBA/2 mice. Mech Ageing Dev. 2010; 131:111–18. 10.1016/j.mad.2009.12.008 20064544

[22] Rebrin I, Forster MJ, Sohal RS. Association between life-span extension by caloric restriction and thiol redox state in two different strains of mice. Free Radic Biol Med. 2011; 51:225–33. 10.1016/j.freeradbiomed.2011.04.006 21530646 PMC3109181

[23] Boldrin L, Ross JA, Whitmore C, Doreste B, Beaver C, Eddaoudi A, Pearce DJ, Morgan JE. The effect of calorie restriction on mouse skeletal muscle is sex, strain and time-dependent. Sci Rep. 2017; 7:5160. 10.1038/s41598-017-04896-y 28698572 PMC5505993

[24] Sohal RS, Ferguson M, Sohal BH, Forster MJ. Life span extension in mice by food restriction depends on an energy imbalance. J Nutr. 2009; 139:533–39. 10.3945/jn.108.100313 19141702 PMC2646218

[25] Liao CY, Rikke BA, Johnson TE, Gelfond JA, Diaz V, Nelson JF. Fat maintenance is a predictor of the murine lifespan response to dietary restriction. Aging Cell. 2011; 10:629–39. 10.1111/j.1474-9726.2011.00702.x 21388497 PMC3685291

[26] Mitchell SJ, Madrigal-Matute J, Scheibye-Knudsen M, Fang E, Aon M, González-Reyes JA, Cortassa S, Kaushik S, Gonzalez-Freire M, Patel B, Wahl D, Ali A, Calvo-Rubio M, et al. Effects of sex, strain, and energy intake on hallmarks of aging in mice. Cell Metab. 2016; 23:1093–112. 10.1016/j.cmet.2016.05.027 27304509 PMC4911707

[27] de Magalhães JP. Why genes extending lifespan in model organisms have not been consistently associated with human longevity and what it means to translation research. Cell Cycle. 2014; 13:2671–73. 10.4161/15384101.2014.950151 25486354 PMC4614541

[28] Berrigan D, Lavigne JA, Perkins SN, Nagy TR, Barrett JC, Hursting SD. Phenotypic effects of calorie restriction and insulin-like growth factor-1 treatment on body composition and bone mineral density of C57BL/6 mice: implications for cancer prevention. In Vivo. 2005; 19:667–74. 15999532

[29] Fontana L, Villareal DT, Das SK, Smith SR, Meydani SN, Pittas AG, Klein S, Bhapkar M, Rochon J, Ravussin E, Holloszy JO, and CALERIE Study Group. Effects of 2-year calorie restriction on circulating levels of IGF-1, IGF-binding proteins and cortisol in nonobese men and women: a randomized clinical trial. Aging Cell. 2016; 15:22–27. 10.1111/acel.12400 26443692 PMC4717266

[30] Fontana L, Weiss EP, Villareal DT, Klein S, Holloszy JO. Long-term effects of calorie or protein restriction on serum IGF-1 and IGFBP-3 concentration in humans. Aging Cell. 2008; 7:681–87. 10.1111/j.1474-9726.2008.00417.x 18843793 PMC2673798

[31] Phelan JP, Rose MR. Why dietary restriction substantially increases longevity in animal models but won’t in humans. Ageing Res Rev. 2005; 4:339–50. 10.1016/j.arr.2005.06.001 16046282

[32] Lai M, Chandrasekera PC, Barnard ND. You are what you eat, or are you? The challenges of translating high-fat-fed rodents to human obesity and diabetes. Nutr Diabetes. 2014; 4:e135. 10.1038/nutd.2014.30 25198237 PMC4183971

[33] Denayer T, Stöhr T, Van Roy M. Animal models in translational medicine: Validation and prediction. New Horiz Transl Med. 2014; 2:5–11.

[34] Swindell WR, Johnston A, Carbajal S, Han G, Wohn C, Lu J, Xing X, Nair RP, Voorhees JJ, Elder JT, Wang XJ, Sano S, Prens EP, et al. Genome-wide expression profiling of five mouse models identifies similarities and differences with human psoriasis. PLoS One. 2011; 6:e18266. 10.1371/journal.pone.0018266 21483750 PMC3070727

[35] Swindell WR, Michaels KA, Sutter AJ, Diaconu D, Fritz Y, Xing X, Sarkar MK, Liang Y, Tsoi A, Gudjonsson JE, Ward NL. Imiquimod has strain-dependent effects in mice and does not uniquely model human psoriasis. Genome Med. 2017; 9:24. 10.1186/s13073-017-0415-3 28279190 PMC5345243

[36] Seok J, Warren HS, Cuenca AG, Mindrinos MN, Baker HV, Xu W, Richards DR, McDonald-Smith GP, Gao H, Hennessy L, Finnerty CC, López CM, Honari S, et al, and Inflammation and Host Response to Injury, Large Scale Collaborative Research Program. Genomic responses in mouse models poorly mimic human inflammatory diseases. Proc Natl Acad Sci USA. 2013; 110:3507–12. 10.1073/pnas.1222878110 23401516 PMC3587220

[37] Barger JL, Vann JM, Cray NL, Pugh TD, Mastaloudis A, Hester SN, Wood SM, Newton MA, Weindruch R, Prolla TA. Identification of tissue-specific transcriptional markers of caloric restriction in the mouse and their use to evaluate caloric restriction mimetics. Aging Cell. 2017; 16:750–60. 10.1111/acel.12608 28556428 PMC5506434

[38] Capel F, Klimcáková E, Viguerie N, Roussel B, Vítková M, Kováciková M, Polák J, Kovácová Z, Galitzky J, Maoret JJ, Hanácek J, Pers TH, Bouloumié A, et al. Macrophages and adipocytes in human obesity: adipose tissue gene expression and insulin sensitivity during calorie restriction and weight stabilization. Diabetes. 2009; 58:1558–67. 10.2337/db09-0033 19401422 PMC2699855

[39] Mutch DM, Pers TH, Temanni MR, Pelloux V, Marquez-Quiñones A, Holst C, Martinez JA, Babalis D, van Baak MA, Handjieva-Darlenska T, Walker CG, Astrup A, Saris WH, et al, and DiOGenes Project. A distinct adipose tissue gene expression response to caloric restriction predicts 6-mo weight maintenance in obese subjects. Am J Clin Nutr. 2011; 94:1399–409. 10.3945/ajcn.110.006858 22030226

[40] Johansson LE, Danielsson AP, Parikh H, Klintenberg M, Norström F, Groop L, Ridderstråle M. Differential gene expression in adipose tissue from obese human subjects during weight loss and weight maintenance. Am J Clin Nutr. 2012; 96:196–207. 10.3945/ajcn.111.020578 22648723

[41] Nookaew I, Svensson PA, Jacobson P, Jernås M, Taube M, Larsson I, Andersson-Assarsson JC, Sjöström L, Froguel P, Walley A, Nielsen J, Carlsson LM. Adipose tissue resting energy expenditure and expression of genes involved in mitochondrial function are higher in women than in men. J Clin Endocrinol Metab. 2013; 98:E370–78. 10.1210/jc.2012-2764 23264395 PMC3633773

[42] Campbell KL, Foster-Schubert KE, Makar KW, Kratz M, Hagman D, Schur EA, Habermann N, Horton M, Abbenhardt C, Kuan LY, Xiao L, Davison J, Morgan M, et al. Gene expression changes in adipose tissue with diet- and/or exercise-induced weight loss. Cancer Prev Res (Phila). 2013; 6:217–31. 10.1158/1940-6207.CAPR-12-0212 23341572 PMC3738189

[43] Magkos F, Fraterrigo G, Yoshino J, Luecking C, Kirbach K, Kelly SC, de Las Fuentes L, He S, Okunade AL, Patterson BW, Klein S. Effects of moderate and subsequent progressive weight loss on metabolic function and adipose tissue biology in humans with obesity. Cell Metab. 2016; 23:591–601. 10.1016/j.cmet.2016.02.005 26916363 PMC4833627

[44] Vink RG, Roumans NJ, Fazelzadeh P, Tareen SH, Boekschoten MV, van Baak MA, Mariman EC. Adipose tissue gene expression is differentially regulated with different rates of weight loss in overweight and obese humans. Int J Obes. 2017; 41:309–16. 10.1038/ijo.2016.201 27840413

[45] Bolton J, Montastier E, Carayol J, Bonnel S, Mir L, Marques MA, Astrup A, Saris W, Iacovoni J, Villa-Vialaneix N, Valsesia A, Langin D, Viguerie N. Molecular biomarkers for weight control in obese individuals subjected to a multiphase dietary intervention. J Clin Endocrinol Metab. 2017; 102:2751–61. 10.1210/jc.2016-3997 28482007

[46] Bollepalli S, Kaye S, Heinonen S, Kaprio J, Rissanen A, Virtanen KA, Pietiläinen KH, Ollikainen M. Subcutaneous adipose tissue gene expression and DNA methylation respond to both short- and long-term weight loss. Int J Obes. 2018; 42:412–23. 10.1038/ijo.2017.245 28978976

[47] Swindell WR. Genes and gene expression modules associated with caloric restriction and aging in the laboratory mouse. BMC Genomics. 2009; 10:585. 10.1186/1471-2164-10-585 19968875 PMC2795771

[48] Plank M, Wuttke D, van Dam S, Clarke SA, de Magalhães JP. A meta-analysis of caloric restriction gene expression profiles to infer common signatures and regulatory mechanisms. Mol Biosyst. 2012; 8:1339–49. 10.1039/c2mb05255e 22327899

[49] Chernoff H. The use of faces to represent points in k-dimensional space graphically. J Am Stat Assoc. 1973; 68:361–68. 10.1080/01621459.1973.10482434

[50] Noyan H, El-Mounayri O, Isserlin R, Arab S, Momen A, Cheng HS, Wu J, Afroze T, Li RK, Fish JE, Bader GD, Husain M. Cardioprotective signature of short-term caloric restriction. PLoS One. 2015; 10:e0130658. 10.1371/journal.pone.0130658 26098549 PMC4476723

[51] Piper MD, Bartke A. Diet and aging. Cell Metab. 2008; 8:99–104. 10.1016/j.cmet.2008.06.012 18680711

[52] Kanehisa M, Sato Y, Kawashima M, Furumichi M, Tanabe M. KEGG as a reference resource for gene and protein annotation. Nucleic Acids Res. 2016; 44:D457–62. 10.1093/nar/gkv1070 26476454 PMC4702792

[53] Shanley DP, Kirkwood TB. Caloric restriction does not enhance longevity in all species and is unlikely to do so in humans. Biogerontology. 2006; 7:165–68. 10.1007/s10522-006-9006-1 16858629

[54] Chusyd DE, Wang D, Huffman DM, Nagy TR. Relationships between rodent white adipose fat pads and human white adipose fat depots. Front Nutr. 2016; 3:10. 10.3389/fnut.2016.00010 27148535 PMC4835715

[55] Tran TT, Yamamoto Y, Gesta S, Kahn CR. Beneficial effects of subcutaneous fat transplantation on metabolism. Cell Metab. 2008; 7:410–20. 10.1016/j.cmet.2008.04.004 18460332 PMC3204870

[56] Neel JV. Diabetes mellitus: a “thrifty” genotype rendered detrimental by “progress”? Am J Hum Genet. 1962; 14:353–62. 13937884 PMC1932342

[57] Chakravarthy MV, Booth FW. Eating, exercise, and “thrifty” genotypes: connecting the dots toward an evolutionary understanding of modern chronic diseases. J Appl Physiol (1985). 2004; 96:3–10. 10.1152/japplphysiol.00757.2003 14660491

[58] Saetrum Opgaard O, Wang PH. IGF-I is a matter of heart. Growth Horm IGF Res. 2005; 15:89–94. 10.1016/j.ghir.2005.02.002 15809013

[59] Ren J, Samson WK, Sowers JR. Insulin-like growth factor I as a cardiac hormone: physiological and pathophysiological implications in heart disease. J Mol Cell Cardiol. 1999; 31:2049–61. 10.1006/jmcc.1999.1036 10591031

[60] Sung MM, Soltys CL, Masson G, Boisvenue JJ, Dyck JR. Improved cardiac metabolism and activation of the RISK pathway contributes to improved post-ischemic recovery in calorie restricted mice. J Mol Med (Berl). 2011; 89:291–302. 10.1007/s00109-010-0703-5 21140129

[61] Glusman G, Yanai I, Rubin I, Lancet D. The complete human olfactory subgenome. Genome Res. 2001; 11:685–702. 10.1101/gr.171001 11337468

[62] Zhang X, Firestein S. The olfactory receptor gene superfamily of the mouse. Nat Neurosci. 2002; 5:124–33. 11802173 10.1038/nn800

[63] Parmentier M, Libert F, Schurmans S, Schiffmann S, Lefort A, Eggerickx D, Ledent C, Mollereau C, Gérard C, Perret J, Grootegoed A, Vassart G. Expression of members of the putative olfactory receptor gene family in mammalian germ cells. Nature. 1992; 355:453–55. 10.1038/355453a0 1370859

[64] Drutel G, Arrang JM, Diaz J, Wisnewsky C, Schwartz K, Schwartz JC. Cloning of OL1, a putative olfactory receptor and its expression in the developing rat heart. Receptors Channels. 1995; 3:33–40. 8589991

[65] Kang N, Koo J. Olfactory receptors in non-chemosensory tissues. BMB Rep. 2012; 45:612–22. 10.5483/BMBRep.2012.45.11.232 23186999 PMC4133803

[66] Yan L, Park JY, Dillinger JG, De Lorenzo MS, Yuan C, Lai L, Wang C, Ho D, Tian B, Stanley WC, Auwerx J, Vatner DE, Vatner SF. Common mechanisms for calorie restriction and adenylyl cyclase type 5 knockout models of longevity. Aging Cell. 2012; 11:1110–20. 10.1111/acel.12013 23020244 PMC3646327

[67] Apfeld J, Kenyon C. Regulation of lifespan by sensory perception in Caenorhabditis elegans. Nature. 1999; 402:804–09. 10.1038/45544 10617200

[68] Libert S, Zwiener J, Chu X, Vanvoorhies W, Roman G, Pletcher SD. Regulation of Drosophila life span by olfaction and food-derived odors. Science. 2007; 315:1133–37. 10.1126/science.1136610 17272684

[69] Chen Z, Zhao H, Fu N, Chen L. The diversified function and potential therapy of ectopic olfactory receptors in non-olfactory tissues. J Cell Physiol. 2018; 233:2104–15. 10.1002/jcp.25929 28338216

[70] Jovancevic N, Dendorfer A, Matzkies M, Kovarova M, Heckmann JC, Osterloh M, Boehm M, Weber L, Nguemo F, Semmler J, Hescheler J, Milting H, Schleicher E, et al. Medium-chain fatty acids modulate myocardial function via a cardiac odorant receptor. Basic Res Cardiol. 2017; 112:13. 10.1007/s00395-017-0600-y 28116519 PMC5258789

[71] Jahromi MM. Haplotype specific alteration of diabetes MHC risk by olfactory receptor gene polymorphism. Autoimmun Rev. 2012; 12:270–74. 10.1016/j.autrev.2012.05.001 22579560

[72] Shepard BD, Cheval L, Peterlin Z, Firestein S, Koepsell H, Doucet A, Pluznick JL. A Renal olfactory receptor aids in kidney glucose handling. Sci Rep. 2016; 6:35215. 10.1038/srep35215 27739476 PMC5064317

[73] Sackmann-Sala L, Berryman DE, Munn RD, Lubbers ER, Kopchick JJ. Heterogeneity among white adipose tissue depots in male C57BL/6J mice. Obesity (Silver Spring). 2012; 20:101–11. 10.1038/oby.2011.235 21779095 PMC3666351

[74] Faulks SC, Turner N, Else PL, Hulbert AJ. Calorie restriction in mice: effects on body composition, daily activity, metabolic rate, mitochondrial reactive oxygen species production, and membrane fatty acid composition. J Gerontol A Biol Sci Med Sci. 2006; 61:781–94. 10.1093/gerona/61.8.781 16912094

[75] Brzęk P, Gębczyński AK, Książek A, Konarzewski M. Effect of calorie restriction on spontaneous physical activity and body mass in mice divergently selected for basal metabolic rate (BMR). Physiol Behav. 2016; 161:116–22. 10.1016/j.physbeh.2016.04.022 27090226

[76] Redman LM, Heilbronn LK, Martin CK, de Jonge L, Williamson DA, Delany JP, Ravussin E, and Pennington CALERIE Team. Metabolic and behavioral compensations in response to caloric restriction: implications for the maintenance of weight loss. PLoS One. 2009; 4:e4377. 10.1371/journal.pone.0004377 19198647 PMC2634841

[77] Martin CK, Heilbronn LK, de Jonge L, DeLany JP, Volaufova J, Anton SD, Redman LM, Smith SR, Ravussin E. Effect of calorie restriction on resting metabolic rate and spontaneous physical activity. Obesity (Silver Spring). 2007; 15:2964–73. 10.1038/oby.2007.354 18198305

[78] Swindell WR. Meta-analysis of 29 experiments evaluating the effects of rapamycin on life span in the laboratory mouse. J Gerontol A Biol Sci Med Sci. 2017; 72:1024–32. 27519886 10.1093/gerona/glw153

[79] Widdowson EM. The response of the sexes to nutritional stress. Proc Nutr Soc. 1976; 35:175–80. 10.1079/PNS19760030 823551

[80] Wiedmer P, Boschmann M, Klaus S. Gender dimorphism of body mass perception and regulation in mice. J Exp Biol. 2004; 207:2859–66. 10.1242/jeb.01120 15235014

[81] Sheng Y, Lv S, Huang M, Lv Y, Yu J, Liu J, Tang T, Qi H, Di W, Ding G. Opposing effects on cardiac function by calorie restriction in different-aged mice. Aging Cell. 2017; 16:1155–67. 10.1111/acel.12652 28799249 PMC5595678

[82] Mattison JA, Colman RJ, Beasley TM, Allison DB, Kemnitz JW, Roth GS, Ingram DK, Weindruch R, de Cabo R, Anderson RM. Caloric restriction improves health and survival of rhesus monkeys. Nat Commun. 2017; 8:14063. 10.1038/ncomms14063 28094793 PMC5247583

[83] Most J, Tosti V, Redman LM, Fontana L. Calorie restriction in humans: an update. Ageing Res Rev. 2017; 39:36–45. 10.1016/j.arr.2016.08.005 27544442 PMC5315691

[84] Zeiss CJ, Johnson LK. Bridging the gap between reproducibility and translation: data resources and approaches. ILAR J. 2017; 58:1–3. 10.1093/ilar/ilx017 28586416

[85] Irizarry RA, Bolstad BM, Collin F, Cope LM, Hobbs B, Speed TP. Summaries of Affymetrix GeneChip probe level data. Nucleic Acids Res. 2003; 31:e15. 10.1093/nar/gng015 12582260 PMC150247

[86] Li H, Zhu D, Cook M. A statistical framework for consolidating “sibling” probe sets for Affymetrix GeneChip data. BMC Genomics. 2008; 9:188. 10.1186/1471-2164-9-188 18435860 PMC2397416

[87] Bolstad BM, Collin F, Brettschneider J, Simpson K, Cope L, Irizarry RA, Speed TP. (2005). Quality assessment of Affymetrix GeneChip Data. In: Gentleman R, Carey V, Huber W, Irizarry RA and Dudoit S, eds. Bioinformatics and Computational Biology Solutions using R and Bioconductor. (New York, NY: Springer).

[88] Liu WM, Mei R, Di X, Ryder TB, Hubbell E, Dee S, Webster TA, Harrington CA, Ho MH, Baid J, Smeekens SP. Analysis of high density expression microarrays with signed-rank call algorithms. Bioinformatics. 2002; 18:1593–99. 10.1093/bioinformatics/18.12.1593 12490443

[89] Smyth GK. Linear models and empirical bayes methods for assessing differential expression in microarray experiments. Stat Appl Genet Mol Biol. 2004; 3:e3. 10.2202/1544-6115.1027 16646809

[90] Benjamini Y, Hochberg Y. Controlling the false discovery rate: a powerful and practical approach to multiple testing. J R Stat Soc B. 1995; 57:289–300.

[91] Allanach K, Mengel M, Einecke G, Sis B, Hidalgo LG, Mueller T, Halloran PF. Comparing microarray versus RT-PCR assessment of renal allograft biopsies: similar performance despite different dynamic ranges. Am J Transplant. 2008; 8:1006–15. 10.1111/j.1600-6143.2008.02199.x 18416738

[92] DerSimonian R, Laird N. Meta-analysis in clinical trials. Control Clin Trials. 1986; 7:177–88. 10.1016/0197-2456(86)90046-2 3802833

