# Supplementary material for: Transcriptional profiling identifies strain-specific effects of caloric restriction and opposite responses in human and mouse white adipose tissue
Source: Aging (Albany NY). 2018 Apr 29;10(4):701–46. doi: 10.18632/aging.101424 (PMC5940131; doi:10.18632/aging.101424)
Supplement: Supplementary File [file aging-10-101424-s001.pdf]

SUPPLEMENTARY MATERIAL

Table S1. Dietary allowance and body weights. The table lists the amount of food provided weekly to each strain (8 – 22 weeks of age). The final columns list average body weights in CTL and CR mice at 22 weeks of age. For the CR treatment (columns 4 and 6), the percent reduction relative to CTL mice is given in parentheses.

Strain	Weeks	Food (g) / Week		Week 22 body weight (g)†	
		CTL	CR (%)	CTL	CR
C57BL6/J	8-14	24	18 (25%)	30.6	23.6 (23%)
	14-22	24	14 (42%)‡		
Balbc/J	8-22	31¶	24 (23%)¶	28.2	27.1 (4%)
C3H/HeJ	8-22	24	18 (25%)	33.7	28.4 (16%)
129S1/SvImJ	8-14	24	18 (25%)	28.3	23.7 (16%)
	14-22	24	14 (42%)‡		
CBA/J	8-22	24	18 (25%)	36.0	26.5 (26%)
DBA/2J	8-22	24	18 (25%)	31.4	25.0 (20%)
B6C3F1/J	8-22	24	18 (25%)	37.5	28.4 (24%)

†Week 22 average body weights were estimated from Figure S1 from Barger et al. 2017 (Aging Cell 16: 750-760). Body weight for each strain and treatment was extracted using the DigitizeIt software (https://www.digitizeit.de/).

‡In weeks 14-22, the amount of food was adjusted in CR-fed C57BL6/J and 129S1/SvImJ mice to provide a total weight loss more comparable to other strains (see Barger et al. 2017; Aging Cell 16: 750-760).

¶Balbc/J exhibited excessive weight loss in provided the CR diet of 18 g/week. The total allowance was therefore increased in CTL and CR treatments (see Barger et al. 2017; Aging Cell 16: 750-760).

Table S2. List of 28 experiments incorporated into human scWAT microarray meta-analysis. The table lists 28 experiments in which microarrays were used to evaluate gene expression in periumbilical scWAT of humans before and after CR. The ID provided for each experiment lists the Gene Expression Omnibus series identifier, sex of subjects (M = male, F = female, B = both male and female), and the CR duration (w = weeks, m = months, y = years). The second column lists the PubMed identifier for the original research article describing the CR protocol and generation of samples (PMID). The number of subjects evaluated in each experiment is indicated (column 3) as well as the number of genes for which gene expression measurements were obtained and incorporated into analyses (column 4; excludes 15% of genes with lowest expression in each experiment). The final columns lists the GEO sample identifiers associated with baseline and post-CR treatments. The ordering of samples in the final columns is matched by subject.

Experiment ID	PMID	No. Subjects	No. Genes	Pre-CR (baseline) samples	Post-CR samples
GSE11975-F-4w	19401422	8	14481	N/A (two-color microarrays were utilized with material from pre- and post-intervention samples hybridized to the same arrays)	GSM302948, GSM302950, GSM302952, GSM302954, GSM302956, GSM302958, GSM302960, GSM302962
GSE11975-F-7m	19401422	8	14481	N/A (two-color microarrays were utilized with material from pre- and post-intervention samples hybridized to the same arrays)	GSM302980, GSM302982, GSM302984, GSM302986, GSM302988, GSM302990, GSM302992, GSM302994
GSE24432-F-8w	22030226	40	11757	GSM602072, GSM602074, GSM602076, GSM602078, GSM602080, GSM602082, GSM602084, GSM602086, GSM602088, GSM602090, GSM602092, GSM602094, GSM602096, GSM602098, GSM602100, GSM602102, GSM602104, GSM602106, GSM602108, GSM602110, GSM602112, GSM602114, GSM602116, GSM602118, GSM602120, GSM602122, GSM602124, GSM602126, GSM602128, GSM602130, GSM602132, GSM602134, GSM602136, GSM602138, GSM602140, GSM602142, GSM602144, GSM602146, GSM602148, GSM602150	GSM602073, GSM602075, GSM602077, GSM602079, GSM602081, GSM602083, GSM602085, GSM602087, GSM602089, GSM602091, GSM602093, GSM602095, GSM602097, GSM602099, GSM602101, GSM602103, GSM602105, GSM602107, GSM602109, GSM602111, GSM602113, GSM602115, GSM602117, GSM602119, GSM602121, GSM602123, GSM602125, GSM602127, GSM602129, GSM602131, GSM602133, GSM602135, GSM602137, GSM602139, GSM602141, GSM602143, GSM602145, GSM602147, GSM602149, GSM602151
SE35411-M-3m	22648723	3	18520	GSM867716, GSM867720, GSM867723	GSM867725, GSM867728, GSM867731
GSE35411-M-9m	22648723	3	18562	GSM867716, GSM867720, GSM867723	GSM867733, GSM867737, GSM867740
GSE35411-F-3m	22648723	5	18531	GSM867722, GSM867724, GSM867718, GSM867717, GSM867721	GSM867730, GSM867732, GSM867727, GSM867726, GSM867729

GSE35411-F-9m	22648723	6	18660	GSM867722, GSM867724, GSM867719, GSM867718, GSM867717, GSM867721	GSM867739, GSM867741, GSM867736, GSM867735, GSM867734, GSM867738
GSE35710-M-4m	23264395	18	11324	GSM873708, GSM873710, GSM873712, GSM873714, GSM873718, GSM873720, GSM873722, GSM873724, GSM873726, GSM873728, GSM873730, GSM873732, GSM873734, GSM873736, GSM873738, GSM873740, GSM873742, GSM873744	GSM873709, GSM873711, GSM873713, GSM873715, GSM873719, GSM873721, GSM873723, GSM873725, GSM873727, GSM873729, GSM873731, GSM873733, GSM873735, GSM873737, GSM873739, GSM873741, GSM873743, GSM873745
GSE35710-F-4m	23264395	6	11305	GSM873700, GSM873702, GSM873704, GSM873706, GSM873716, GSM873746	GSM873701, GSM873703, GSM873705, GSM873707, GSM873717, GSM873747
GSE43471-F-6m-a	23341572	9	18854	GSM1063185, GSM1063193, GSM1063197, GSM1063209, GSM1063221, GSM1063233, GSM1063245, GSM1063257, GSM1063271	GSM1063186, GSM1063194, GSM1063198, GSM1063210, GSM1063222, GSM1063234, GSM1063246, GSM1063258, GSM1063272
GSE43471-F-6m-b	23341572	16	18955	GSM1063189, GSM1063191, GSM1063201, GSM1063203, GSM1063213, GSM1063217, GSM1063225, GSM1063229, GSM1063237, GSM1063241, GSM1063249, GSM1063251, GSM1063261, GSM1063263, GSM1063267, GSM1063275	GSM1063190, GSM1063192, GSM1063202, GSM1063204, GSM1063214, GSM1063218, GSM1063226, GSM1063230, GSM1063238, GSM1063242, GSM1063250, GSM1063252, GSM1063262, GSM1063264, GSM1063268, GSM1063276
GSE70529-B-14w	26916363	9	16185	GSM1808419, GSM1808396, GSM1808431, GSM1808403, GSM1808407, GSM1808411, GSM1808415, GSM1808423, GSM1808427	GSM1808420, GSM1808397, GSM1808400, GSM1808404, GSM1808408, GSM1808412, GSM1808416, GSM1808424, GSM1808428
GSE70529-B-7m	26916363	9	16207	GSM1808419, GSM1808396, GSM1808431, GSM1808403, GSM1808407, GSM1808411, GSM1808415, GSM1808423, GSM1808427	GSM1808398, GSM1808401, GSM1808405, GSM1808409, GSM1808413, GSM1808417, GSM1808421, GSM1808425, GSM1808429
GSE70529-B-10m	26916363	9	16220	GSM1808419, GSM1808396, GSM1808431, GSM1808403, GSM1808407, GSM1808411, GSM1808415, GSM1808423, GSM1808427	GSM1808399, GSM1808402, GSM1808406, GSM1808410, GSM1808414, GSM1808418, GSM1808422, GSM1808426, GSM1808430

GSE77962-M-5w	27840413	11	16263	GSM2062472, GSM2062478, GSM2062518, GSM2062527, GSM2062530, GSM2062539, GSM2062542, GSM2062575, GSM2062578, GSM2062602, GSM2062609	GSM2062473, GSM2062479, GSM2062519, GSM2062528, GSM2062531, GSM2062540, GSM2062543, GSM2062576, GSM2062579, GSM2062603, GSM2062610
GSE77962-M-3m	27840413	10	16273	GSM2062493, GSM2062502, GSM2062512, GSM2062521, GSM2062548, GSM2062590, GSM2062593, GSM2062599, GSM2062605, GSM2062614	GSM2062494, GSM2062503, GSM2062513, GSM2062522, GSM2062549, GSM2062591, GSM2062594, GSM2062600, GSM2062606, GSM2062615
GSE77962-M-9w	27840413	12	16309	GSM2062472, GSM2062478, GSM2062518, GSM2062527, GSM2062530, GSM2062539, GSM2062542, GSM2062575, GSM2062578, GSM2062602, GSM2062609, GSM2062616	GSM2062474, GSM2062480, GSM2062520, GSM2062529, GSM2062532, GSM2062541, GSM2062544, GSM2062577, GSM2062580, GSM2062604, GSM2062611, GSM2062617
GSE77962-M-4m	27840413	9	16256	GSM2062493, GSM2062502, GSM2062512, GSM2062521, GSM2062548, GSM2062590, GSM2062593, GSM2062599, GSM2062612	GSM2062495, GSM2062504, GSM2062514, GSM2062523, GSM2062550, GSM2062592, GSM2062595, GSM2062601, GSM2062613
GSE77962-F-5w	27840413	13	16268	GSM2062466, GSM2062469, GSM2062475, GSM2062496, GSM2062515, GSM2062533, GSM2062536, GSM2062560, GSM2062563, GSM2062569, GSM2062581, GSM2062584, GSM2062587	GSM2062467, GSM2062470, GSM2062476, GSM2062497, GSM2062516, GSM2062534, GSM2062537, GSM2062561, GSM2062564, GSM2062570, GSM2062582, GSM2062585, GSM2062588
GSE77962-F-3m	27840413	14	16239	GSM2062481, GSM2062484, GSM2062487, GSM2062490, GSM2062499, GSM2062509, GSM2062524, GSM2062545, GSM2062551, GSM2062554, GSM2062557, GSM2062566, GSM2062572, GSM2062596	GSM2062482, GSM2062485, GSM2062488, GSM2062491, GSM2062500, GSM2062510, GSM2062525, GSM2062546, GSM2062552, GSM2062555, GSM2062558, GSM2062567, GSM2062573, GSM2062597
GSE77962-F-9w	27840413	14	16257	GSM2062466, GSM2062469, GSM2062475, GSM2062496, GSM2062507, GSM2062515, GSM2062533, GSM2062536, GSM2062560, GSM2062563, GSM2062569, GSM2062581, GSM2062584, GSM2062587	GSM2062468, GSM2062471, GSM2062477, GSM2062498, GSM2062508, GSM2062517, GSM2062535, GSM2062538, GSM2062562, GSM2062565, GSM2062571, GSM2062583, GSM2062586, GSM2062589

GSE77962-F-4m	27840413	14	16289	GSM2062481, GSM2062484, GSM2062487, GSM2062490, GSM2062499, GSM2062509, GSM2062524, GSM2062545, GSM2062551, GSM2062554, GSM2062557, GSM2062566, GSM2062572, GSM2062596	GSM2062483, GSM2062486, GSM2062489, GSM2062492, GSM2062501, GSM2062511, GSM2062526, GSM2062547, GSM2062553, GSM2062556, GSM2062559, GSM2062568, GSM2062574, GSM2062598
GSE95624-M-8m	28482007	6	15817	GSM2519391, GSM2519395, GSM2519397, GSM2519401, GSM2519419, GSM2519421	GSM2519392, GSM2519396, GSM2519398, GSM2519402, GSM2519420, GSM2519422
GSE95624-F-8m	28482007	15	15698	GSM2519393, GSM2519399, GSM2519403, GSM2519405, GSM2519407, GSM2519409, GSM2519411, GSM2519413, GSM2519415, GSM2519417, GSM2519423, GSM2519425, GSM2519427, GSM2519429, GSM2519431	GSM2519394, GSM2519400, GSM2519404, GSM2519406, GSM2519408, GSM2519410, GSM2519412, GSM2519414, GSM2519416, GSM2519418, GSM2519424, GSM2519426, GSM2519428, GSM2519430, GSM2519432
GSE103766-M-5m	28978976	7	18362	GSM2781426, GSM2781429, GSM2781432, GSM2781435, GSM2781439, GSM2781440, GSM2781442	GSM2781445, GSM2781448, GSM2781451, GSM2781454, GSM2781458, GSM2781459, GSM2781461
GSE103766-M-1y	28978976	7	18385	GSM2781426, GSM2781429, GSM2781432, GSM2781435, GSM2781439, GSM2781440, GSM2781442	GSM2781464, GSM2781467, GSM2781470, GSM2781473, GSM2781477, GSM2781478, GSM2781480
GSE103766-F-5m	28978976	12	18447	GSM2781424, GSM2781425, GSM2781427, GSM2781428, GSM2781430, GSM2781431, GSM2781433, GSM2781434, GSM2781436, GSM2781437, GSM2781438, GSM2781441	GSM2781443, GSM2781444, GSM2781446, GSM2781447, GSM2781449, GSM2781450, GSM2781452, GSM2781453, GSM2781455, GSM2781456, GSM2781457, GSM2781460
GSE103766-F-1y	28978976	12	18444	GSM2781424, GSM2781425, GSM2781427, GSM2781428, GSM2781430, GSM2781431, GSM2781433, GSM2781434, GSM2781436, GSM2781437, GSM2781438, GSM2781441	GSM2781462, GSM2781463, GSM2781465, GSM2781466, GSM2781468, GSM2781469, GSM2781471, GSM2781472, GSM2781474, GSM2781475, GSM2781476, GSM2781479

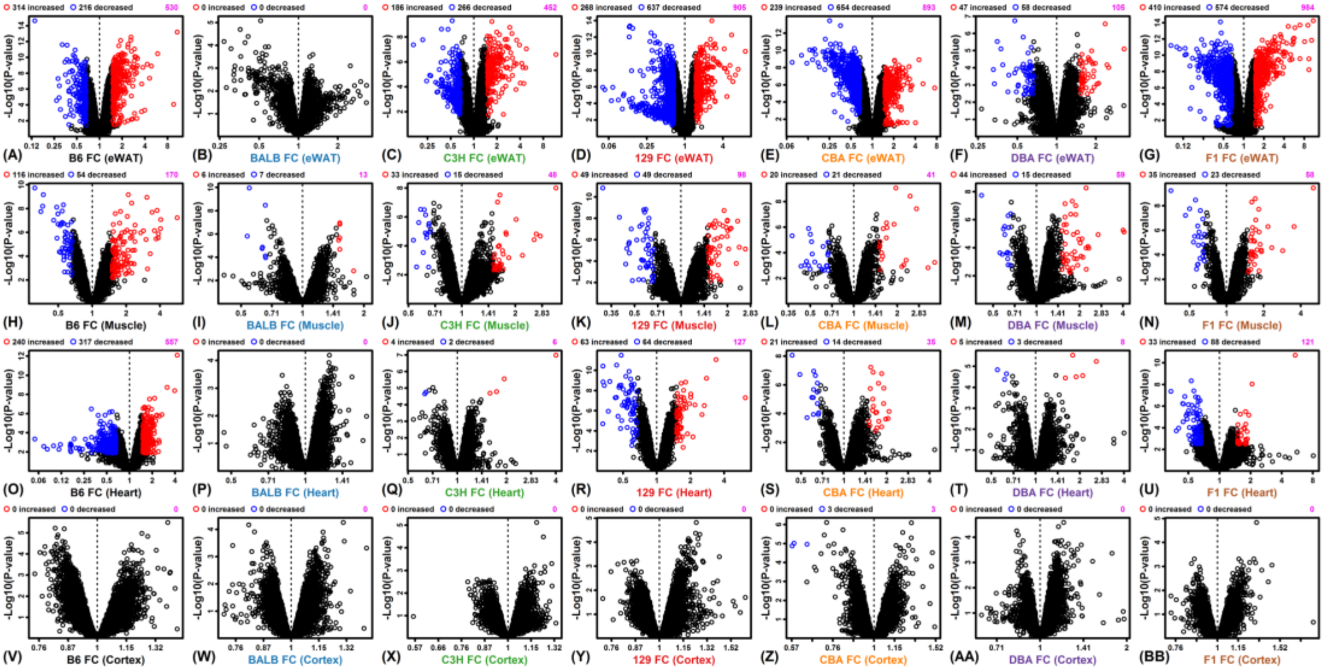

Figure S1. Volcano plots. Differential expression analyses were completed for each of the 28 strain-tissue combinations. Plots compare FC (CR/CTL) and –log10-transformed p-values obtained from linear model tests for differential expression. Red symbols indicate genes significantly increased by CR (FDR < 0.10; FC > 1.50) and blue symbols indicate genes significantly decreased by CR (FDR < 0.10; FC < 0.67). The total number of differentially expressed genes (increased + decreased) is indicated in the upper right (magenta font).

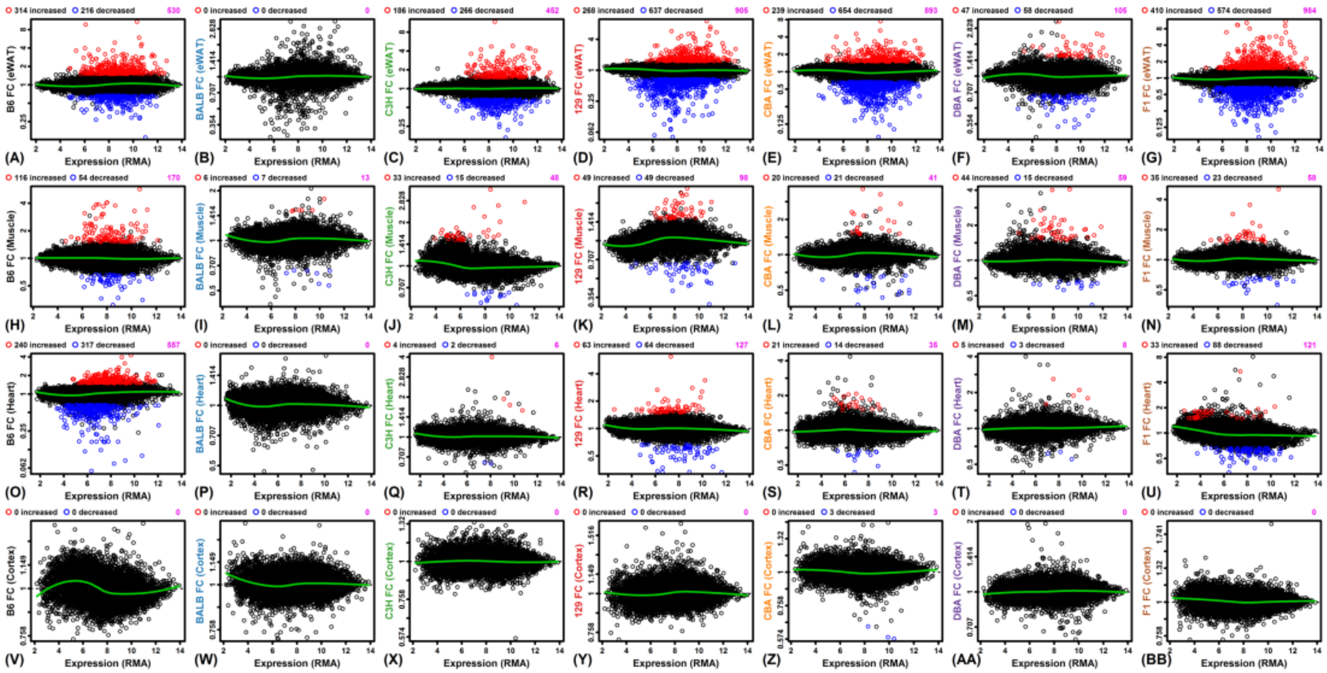

Figure S2. MA plots. Differential expression analyses were completed for each of the 28 strain-tissue combinations. Plots compare FC (CR/CTL) and the average RMA-normalized expression level for each gene. Red symbols indicate genes significantly increased by CR (FDR < 0.10; FC > 1.50) and blue symbols indicate genes significantly decreased by CR (FDR < 0.10; FC < 0.67). The total number of differentially expressed genes (increased + decreased) is indicated in the upper right (magenta font).

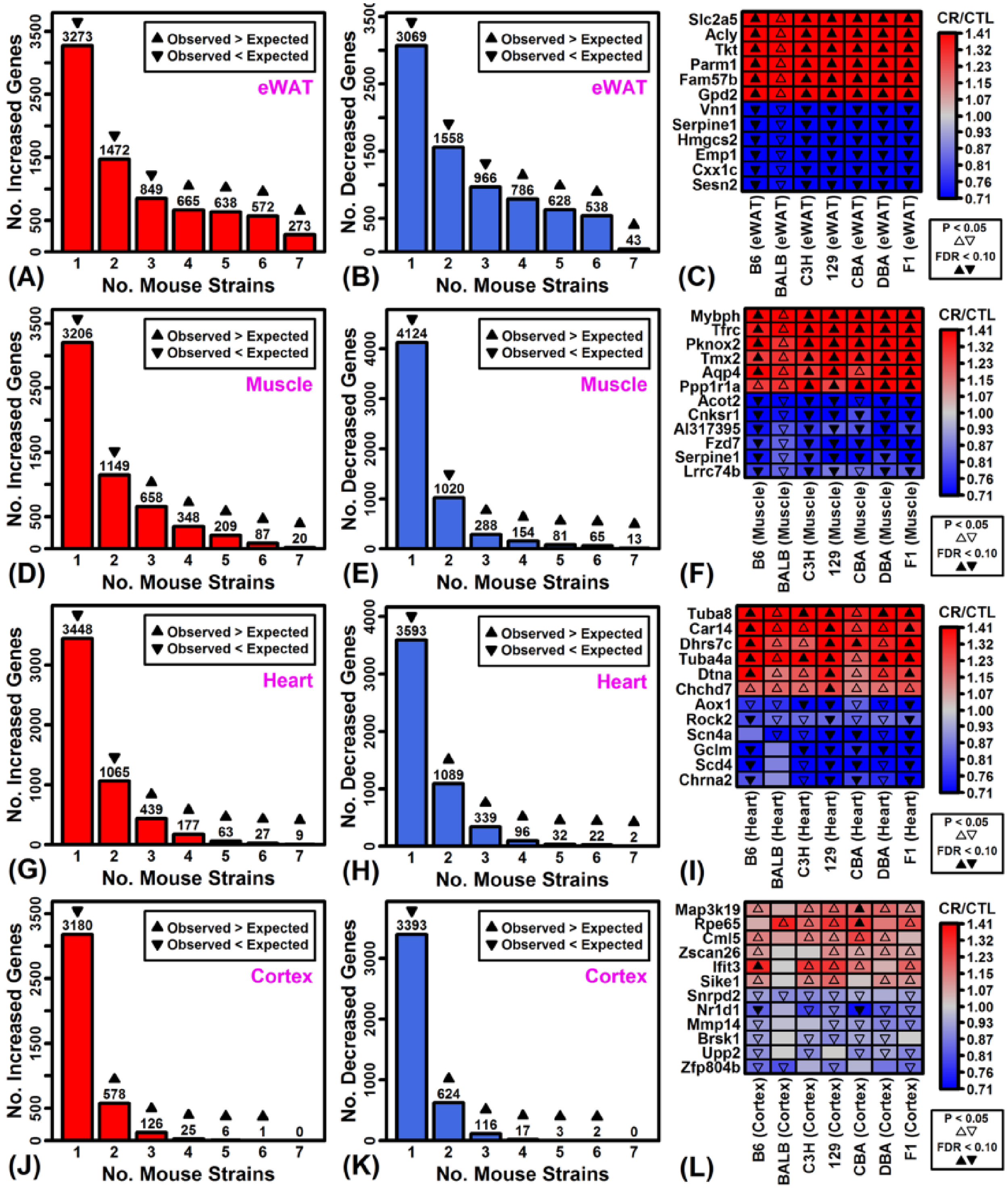

Figure S3. CR gene expression responses shared among multiple strains. (**A, D, G, J**) Number of genes increased by CR in multiple mouse strains at a nominal p-value of 0.05. Up- or down-arrows indicate whether the number of CR-increased genes is significantly larger or smaller than expected ($P < 0.05$; n = 10,000 simulations), given the number of CR-increased genes identified per strain at a p-value threshold of 0.05. (**B, E, H, K**) Number of genes decreased by CR in multiple mouse strains at a nominal p-value of 0.05. Up- or down-arrows indicate whether the number of CR-decreased genes is significantly larger or smaller than expected ($P < 0.05$; n = 10,000 simulations), given the number of CR-decreased genes identified per strain at a p-value threshold of 0.05. (**C, F, I, L**) Genes with the most consistent gene expression responses across the 7 mouse strains.

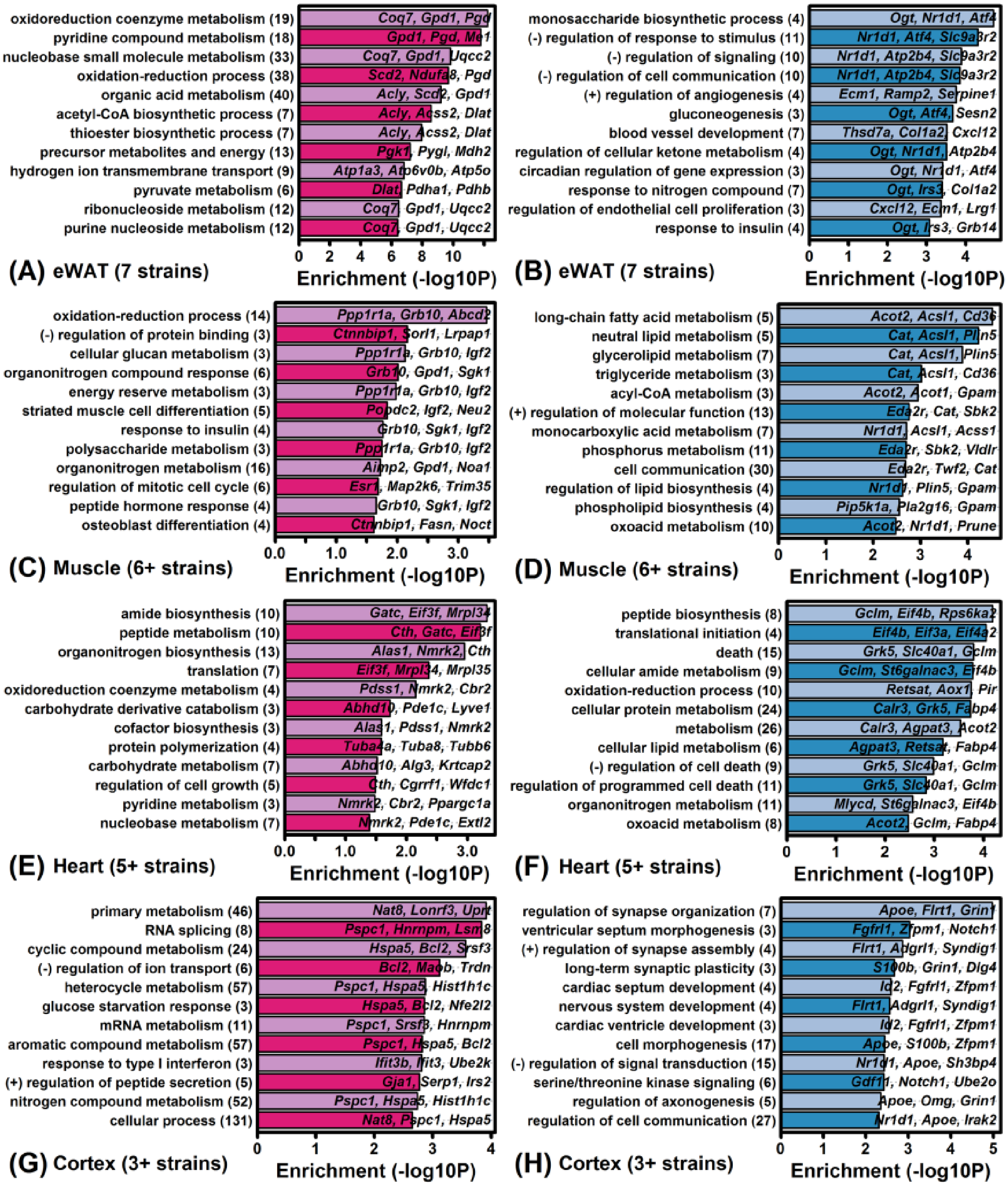

Figure S4. Gene ontology (GO) biological process (BP) terms associated with CR responses shared by multiple mouse strains. (**A, C, E, G**) GO BP terms associated with genes increased by CR in multiple strains (P < 0.05). (**B, D, F, H**) GO BP terms associated with genes decreased by CR in multiple strains (P < 0.05). The top-ranked 12 GO BP terms are listed for each analysis. GO BP term labels are listed in the left margin with the number of associated CR-increased/decreased genes given in parentheses. CR-increased/decreased example genes associated with each term are listed in each figure. The enrichment score (horizontal axis) is calculated as the –log10-transformed p-value from a conditional hypergeometric test for overrepresentation among genes similarly altered in multiple mouse strains.

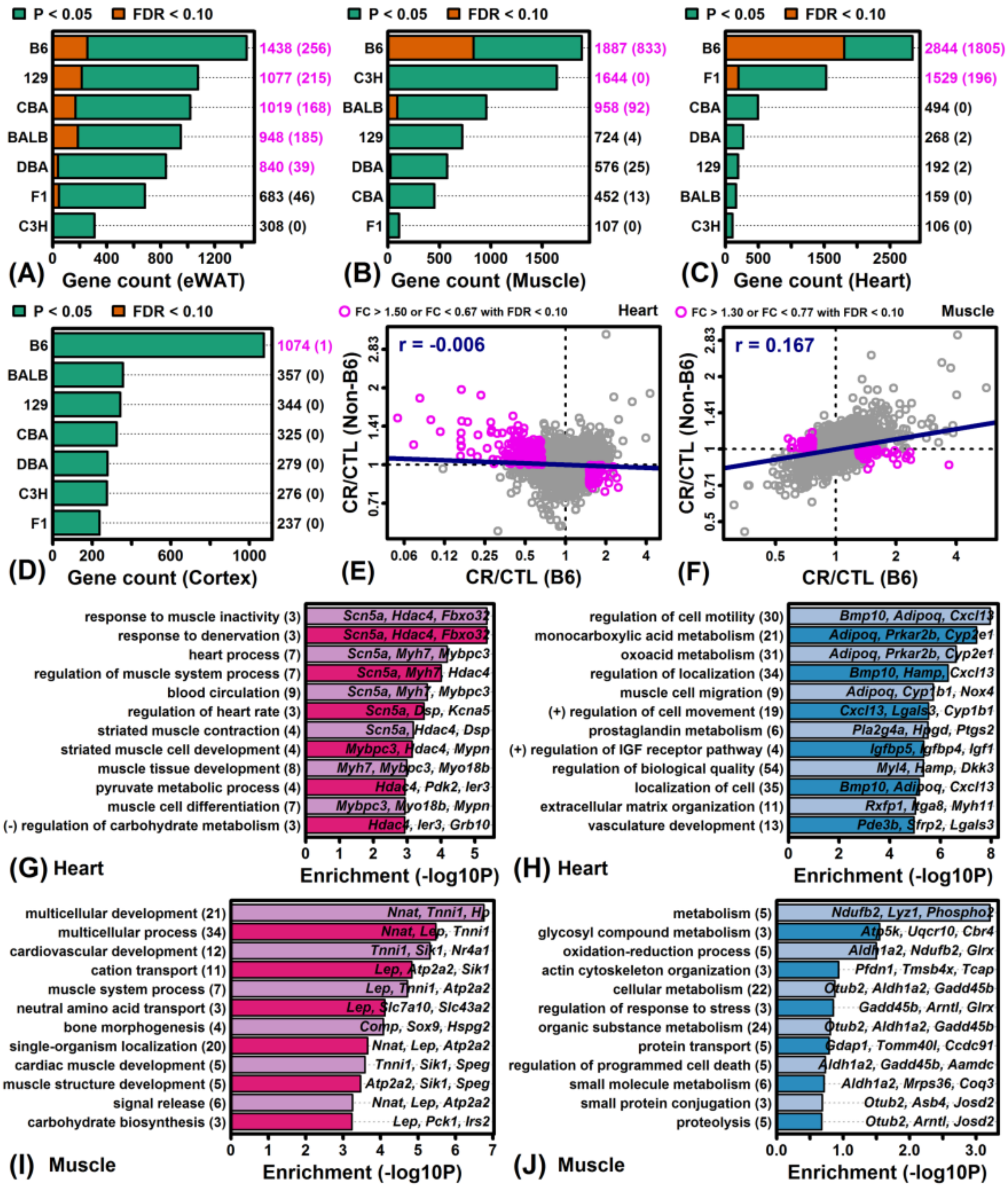

Figure S5. C57BL/6 mice have the largest number of strain-specific responses in each tissue (strain-by-diet interaction effects). (**A-D**) Number of strain-by-diet interaction effects identified with respect to (A) eWAT, (B) muscle, (C) heart and (D) cortex. Strain-by-diet interactions were identified at the indicated significance levels (P < 0.05 or FDR < 0.10) and required to have different FC directions (CR/CTL) in mice from a given strain and other mice from different strains. (**E, F**) FC comparison in B6 and non-B6 mice for (E) heart and (F) muscle. Each point represents a single gene and those altered significantly in heart or muscle are indicated (magenta symbols; upper left: Spearman rank correlation). (**G, H, I, J**) GO BP terms enriched among genes specifically altered by CR in B6 mice (G: CR-increased in B6 heart; H: CR-decreased in B6 heart; I: CR-increased in B6 muscle; J: CR-decreased in B6 muscle). Genes analyzed in (G – J) are shown with magenta symbols in (E) and (F). GO BP term labels are listed in the left margin with the number of associated B6-specific CR-increased/decreased genes given in parentheses. B6-specific CR-increased/decreased example genes associated with each term are listed in each figure. The enrichment score (horizontal axis) is calculated as the –log10-transformed p-value from a conditional hypergeometric test for overrepresentation among B6-specific CR-increased/decreased genes.

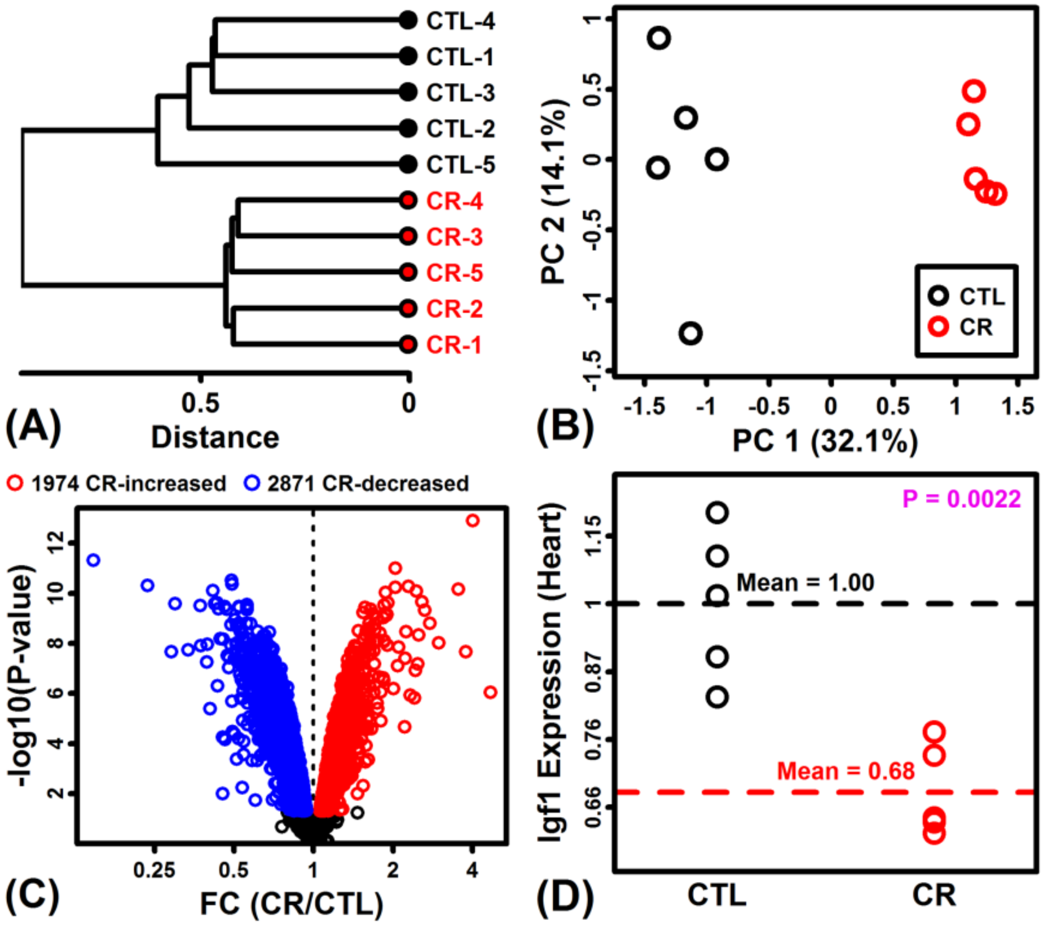

Figure S6. *Igf1* expression is decreased by short-term (1 week) 30% CR in cardiac tissue from 10-12 week old C57BL/6 mice (GSE68646). (**A**) Cluster analysis. DNA microarrays were used to measure gene expression in cardiac tissue from 5 CTL and 5 CR mice (see Noyan et al. 2015, PLoS ONE: 10:e0130658). The 10 samples were clustered based upon the expression of protein-coding genes with detectable expression (Euclidean distance metric; average linkage). (**B**) Principal component plot. The 10 samples are plotted with respect to the first 2 principal components extracted from the normalized expression matrix including the 10 samples and protein-coding genes with detectable expression. (**C**) Volcano plot. Log10-transformed p-values (vertical axis) are plotted against FC estimates (horizontal axis). CR-increased genes with FC > 1.00 and FDR < 0.10 are indicated by red symbols, and CR-decreased genes with FC < 1.00 and FDR < 0.10 are indicated by blue symbols. Differential expression analyses were performed using linear models with moderated t-statistics (R package: limma). (**D**) Insulin-like growth factor 1 (*Igf1*) expression. Microarray-based expression intensities for *Igf1* are plotted for each sample. Expression intensities are normalized to the average expression level of the CTL treatment. The p-value (upper right) was obtained using a two-sample t-test.

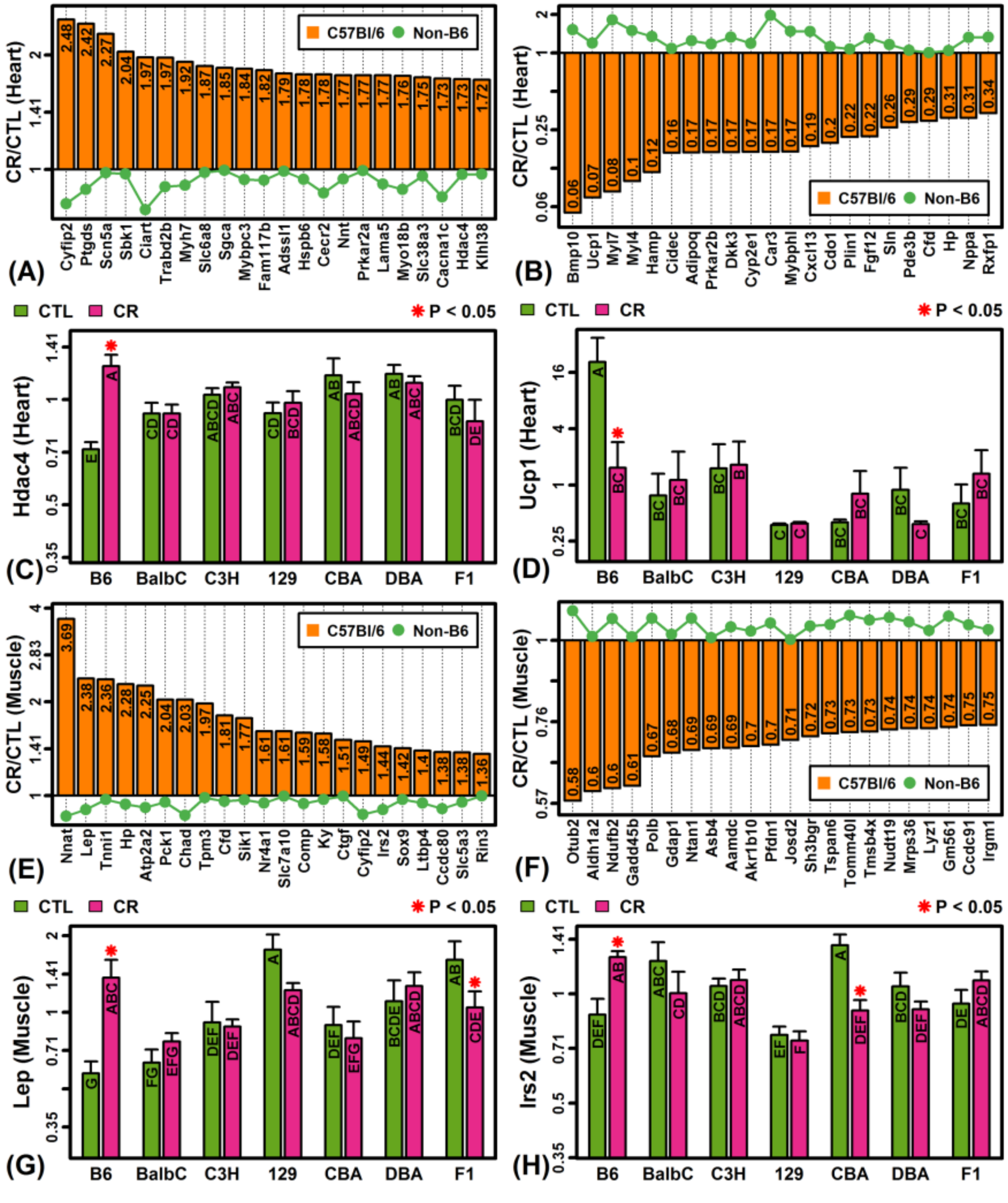

Figure S7. Genes specifically altered by CR in C57BL/6 mice (heart and muscle). (**A**) Genes specifically increased by CR in heart of C57BL/6 mice. (**B**) Genes specifically decreased by CR in heart of C57BL/6 mice. (**C**) Histone deacetylase 4 (Hdac4). (**D**) Uncoupling protein 1 (Ucp1). (**E**) Genes specifically increased by CR in muscle of C57BL/6 mice. (**F**) Genes specifically decreased by CR in muscle of C57BL/6 mice. (**G**) Leptin (Lep). (**H**) Insulin receptor substrate 2 (Irs2). In (A), (B), (E) and (F), the green line denotes the average FC of the 6 non-B6 strains. In (C), (D), (G) and (H), asterisks indicate that the CR treatment differs significantly from the CTL treatment for a given strain ($P < 0.05$). Treatments that share the same letter do not differ significantly ($P < 0.05$, Fisher's least significant difference).

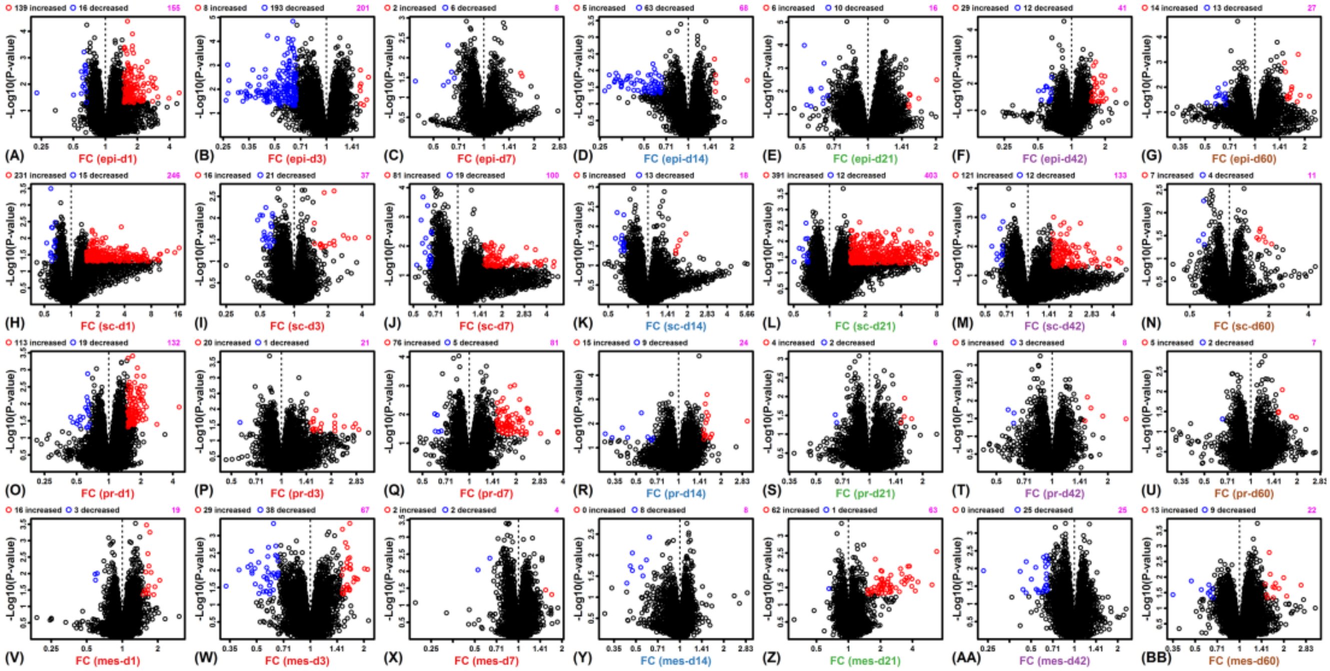

Figure S8. Volcano plots for differential expression analysis of 4 WAT depots from CR- and *ad lib*-fed obese C57BL/6 males (GSE30534). Differential expression analyses were completed for each WAT depot (epi = epididymal; sc = subcutaneous; pr = perirenal; mes = mesenteric) and CR time point (days 1, 3, 7, 14, 21, 42 and 60). Plots compare FC (CR/CTL) and –log10-transformed p-values obtained from linear model tests for differential expression. Red symbols indicate genes increased by CR ($P < 0.05$; $FC > 1.50$) and blue symbols indicate genes decreased by CR ($P < 0.05$; $FC < 0.67$). The total number of genes significantly altered at these thresholds (increased + decreased) is indicated in the upper right (magenta font).

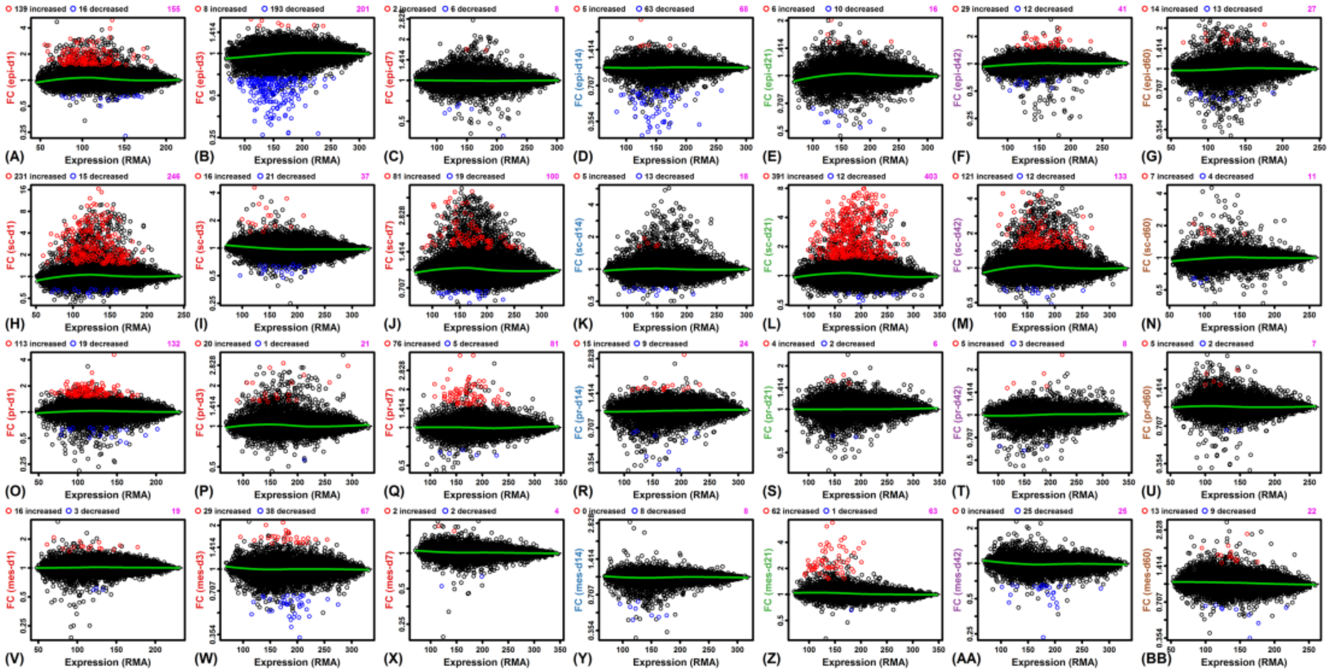

Figure S9. MA plots for differential expression analysis of 4 WAT depots from CR- and *ad lib*-fed obese C57BL/6 males (GSE30534). Differential expression analyses were completed for each WAT depot (epi = epididymal; sc = subcutaneous; pr = perirenal; mes = mesenteric) and CR time point (days 1, 3, 7, 14, 21, 42 and 60). Plots compare FC (CR/CTL) and the average RMA-normalized expression level for each gene. Red symbols indicate genes increased by CR ($P < 0.05$; $FC > 1.50$) and blue symbols indicate genes significantly decreased by CR ($P < 0.05$; $FC < 0.67$). The total number of genes significantly altered at these thresholds (increased + decreased) is indicated in the upper right (magenta font).

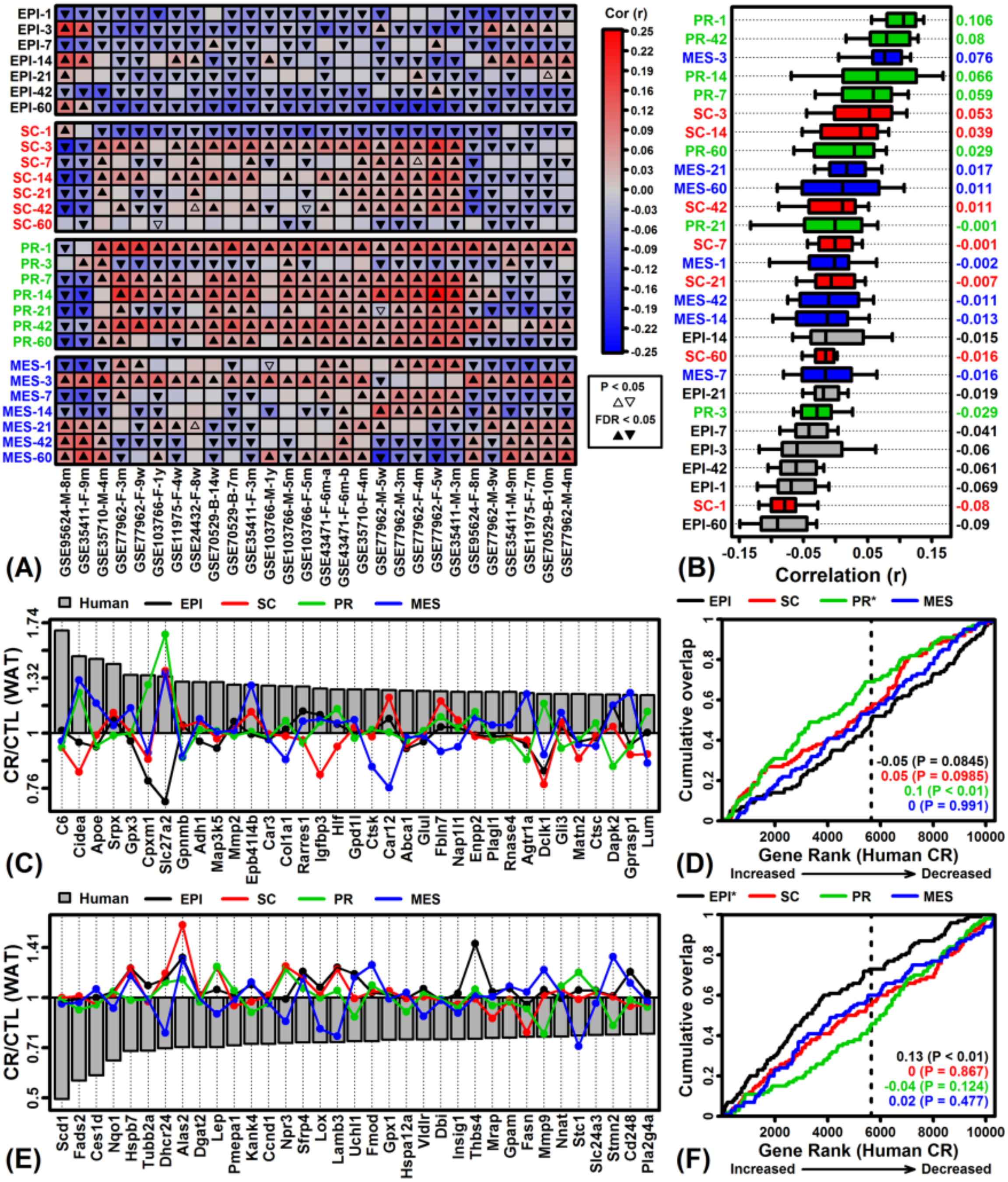

Figure S10. Gene expression responses to CR in human scWAT compared to 4 adipose depots from obese C57BL/6 males (GSE30534). (**A**) Genome-wide spearman rank correlations between fold-change estimates (CR/CTL). Effects of 25% CR in mice fed a high fat diet were evaluated following 1, 3, 7, 14, 21, 42 and 60 days with respect to epididymal (EPI), abdominal subcutaneous (SC), perirenal (PR) and mesenteric (MES) adipose tissue. (**B**) Correlation estimates for each depot and time point. Bars span the middle 50% of correlations for each depot and time point (whiskers: middle 80%; right margin: median correlation). (**C**) FC estimates for genes most strongly increased by CR in human scWAT. Mouse FC estimates are shown for each mouse depot (60 day time point). (**D**) GSEA of top 100 CR-increased genes in each mouse strain. (**E**) FC estimates for genes most strongly decreased by CR in human scWAT. Mouse FC estimates are shown for each mouse depot (60 day time point). (**F**) GSEA of top 100 CR-decreased genes in each mouse strain. In (D) and (F), genes were ranked according to their expression change with CR in humans (horizontal axis) and cumulative overlap was examined with respect to 100 CR-increased/decreased genes from each mouse depot at the 60 day time point (vertical axis) (*P < 0.05, upper margin labels; enrichment statistics with p-values listed in each figure). Positive enrichment statistics indicate significant overlap with respect to genes increased by CR in human scWAT, while negative statistics indicate significant overlap with respect to genes decreased by CR in human scWAT (dashed vertical line: number of CR-increased genes in human, FC > 1.00).

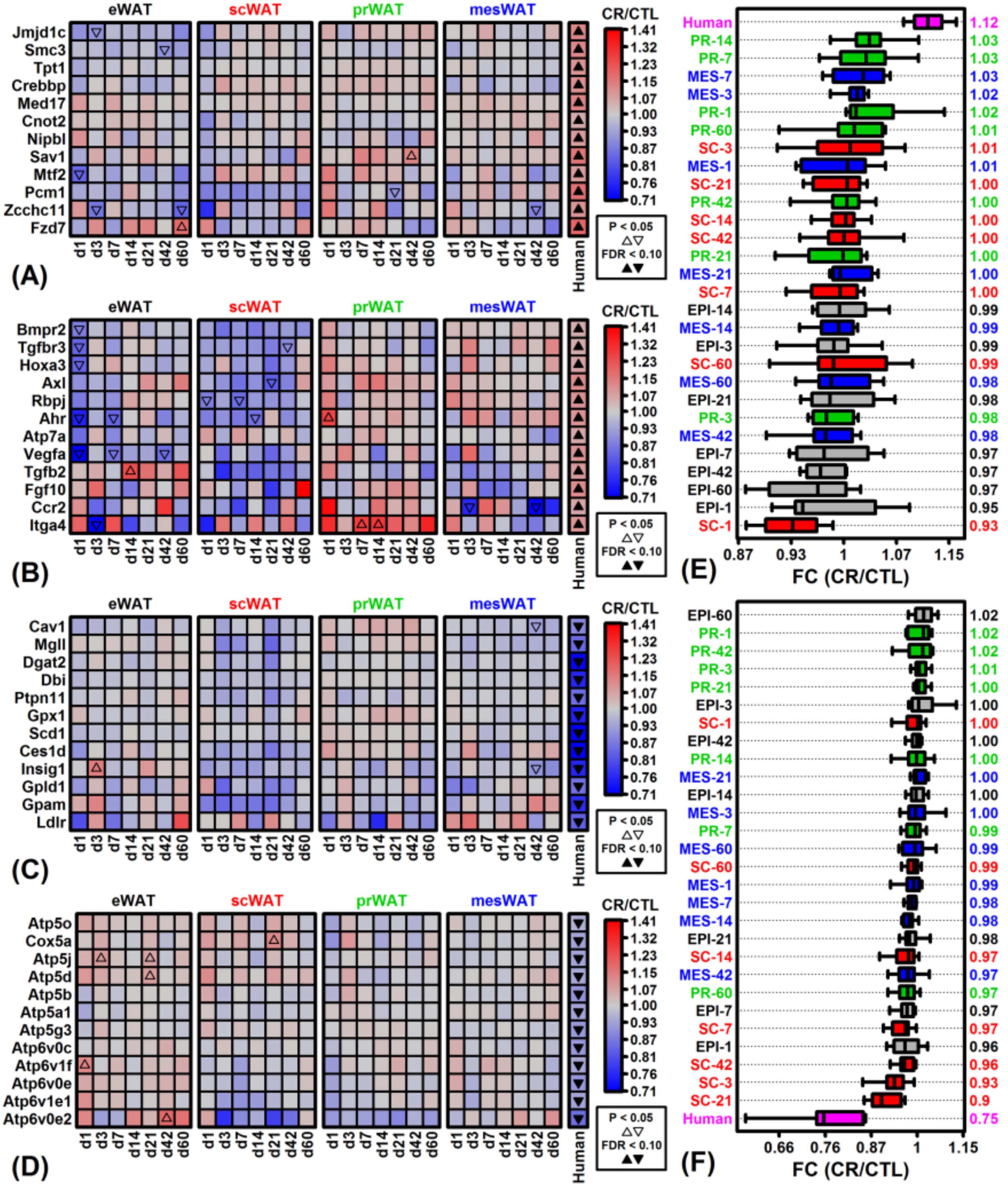

Figure S11. Effects of CR on the expression of genes associated with stem cell maintenance, blood vessel remodeling, lipid metabolism and hydrogen ion transport in 4 WAT depots from obese C57BL/6 males (GSE30534). (**A – D**) CR response heatmaps for genes associated with (A) stem cell population maintenance (GO:0019827), (B) blood vessel remodeling (GO:0001974), (C) neutral lipid metabolism (GO:0006638), and (D) hydrogen ion membrane transport (GO:1902600). CR responses are shown for 4 WAT depots following 1, 3, 7, 14, 21, 42 and 60 days of CR along with the human CR response in scWAT (meta-analysis estimates). Each heatmap shows the 12 genes associated with each GO term most strongly altered by CR in human scWAT. (**E, F**) Median FC (CR/CTL) for genes associated with (E) stem cell population maintenance (GO:0019827) and (F) neutral lipid metabolism (GO:0006638). Bars span the middle 50% of FC estimates (CR/CTL) for each depot and time point (whiskers: middle 80%; right margin: median FC).

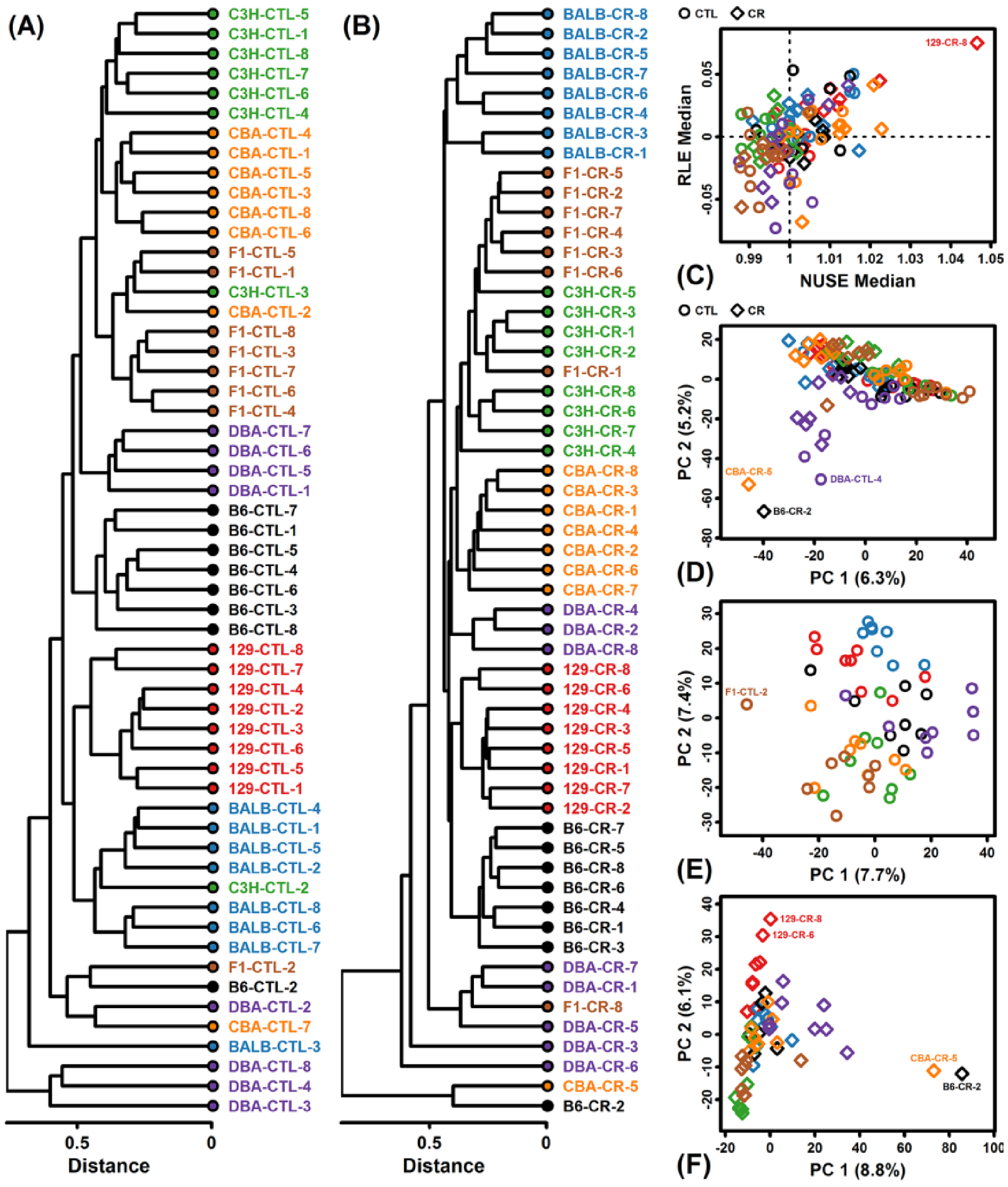

Figure S12. eWAT sample quality control (*n* = 112 samples). (**A**) CTL sample cluster analysis. (**B**) CR sample cluster analysis. In (A) and (B), samples were clustered based upon the Euclidean distance between expression profiles of protein-coding genes (average linkage method). (**C**) Scatterplot comparison between probe-level model NUSE and RLE medians (NUSE: normalized unscaled standard error; RLE: relative log expression). Sample hybridizations with NUSE median differing substantially from 1 or RLE median differing substantially from 0 are potentially of low quality. (**D**) PC scatterplot (CTL + CR samples). (**E**) PC scatterplot (CTL samples only). (**F**) PC scatterplot (CR samples only).

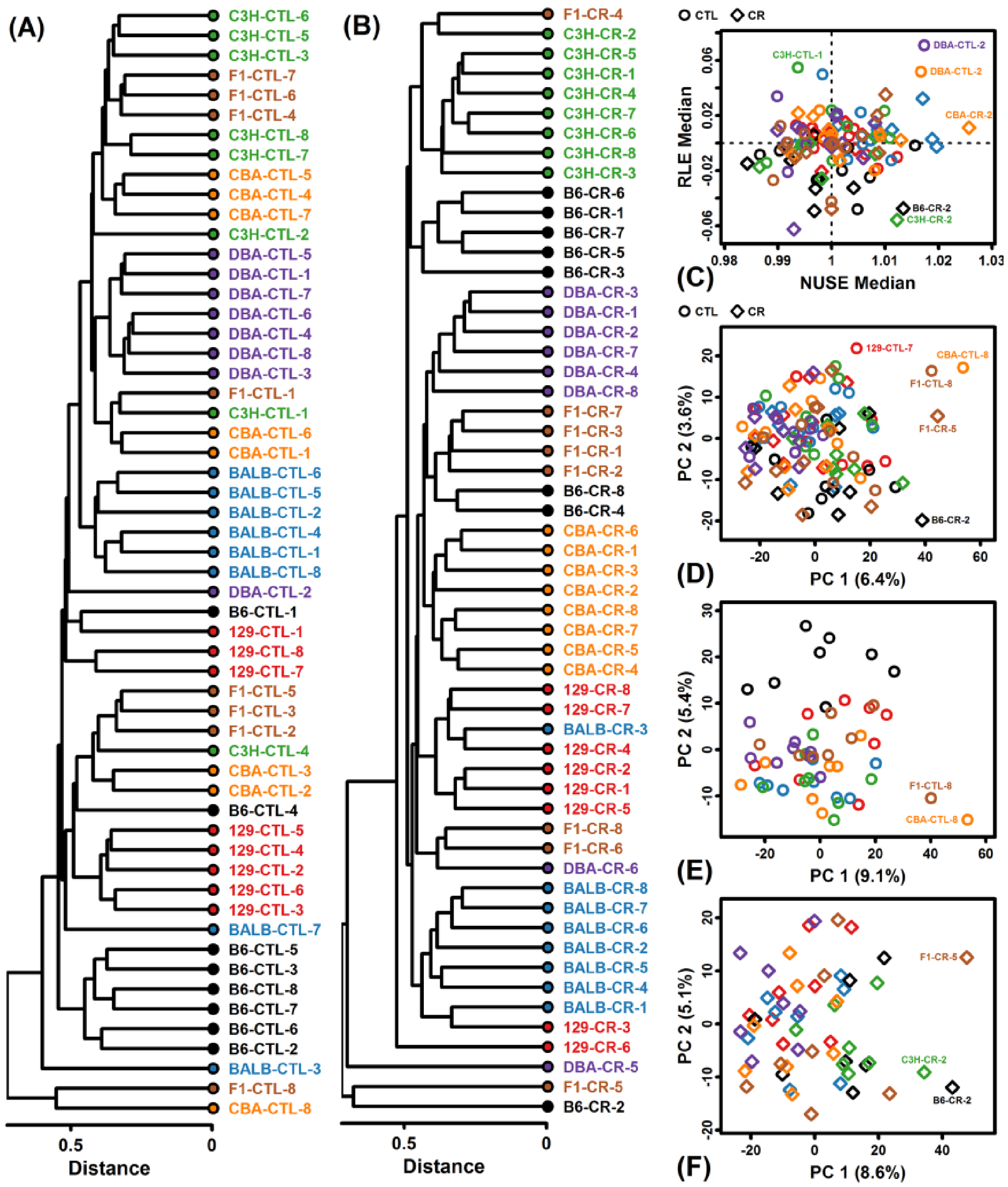

Figure S13. Muscle sample quality control (*n* = 112 samples). (**A**) CTL sample cluster analysis. (**B**) CR sample cluster analysis. In (A) and (B), samples were clustered based upon the Euclidean distance between expression profiles of protein-coding genes (average linkage method). (**C**) Scatterplot comparison between probe-level model NUSE and RLE medians (NUSE: normalized unscaled standard error; RLE: relative log expression). Sample hybridizations with NUSE median differing substantially from 1 or RLE median differing substantially from 0 are potentially of low quality. (**D**) PC scatterplot (CTL + CR samples). (**E**) PC scatterplot (CTL samples only). (**F**) PC scatterplot (CR samples only).

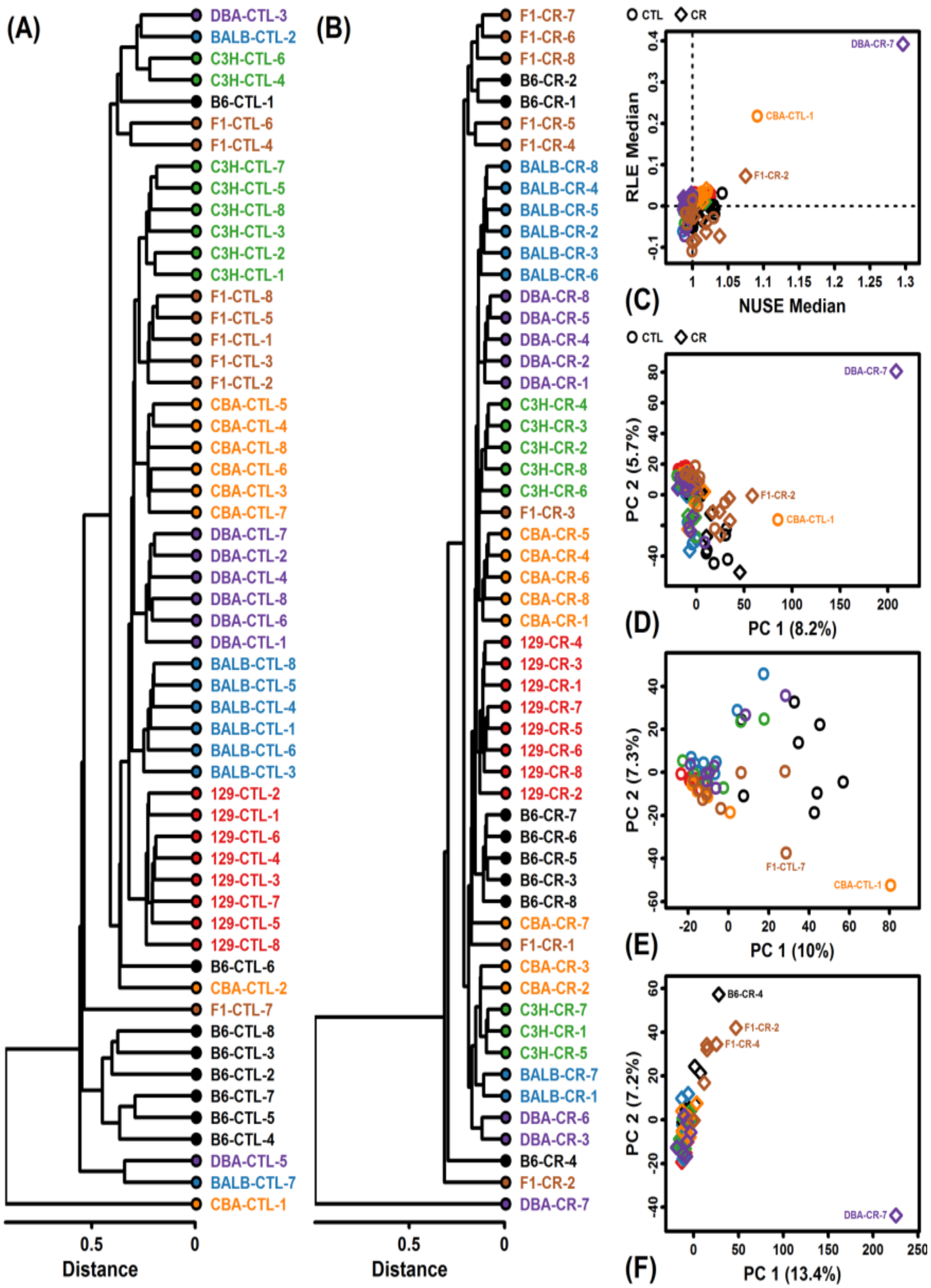

Figure S14. Heart sample quality control (*n* = 112 samples). (**A**) CTL sample cluster analysis. (**B**) CR sample cluster analysis. In (A) and (B), samples were clustered based upon the Euclidean distance between expression profiles of protein-coding genes (average linkage method). (**C**) Scatterplot comparison between probe-level model NUSE and RLE medians (NUSE: normalized unscaled standard error; RLE: relative log expression). Sample hybridizations with NUSE median differing substantially from 1 or RLE median differing substantially from 0 are potentially of low quality. (**D**) PC scatterplot (CTL + CR samples). (**E**) PC scatterplot (CTL samples only). (**F**) PC scatterplot (CR samples only).

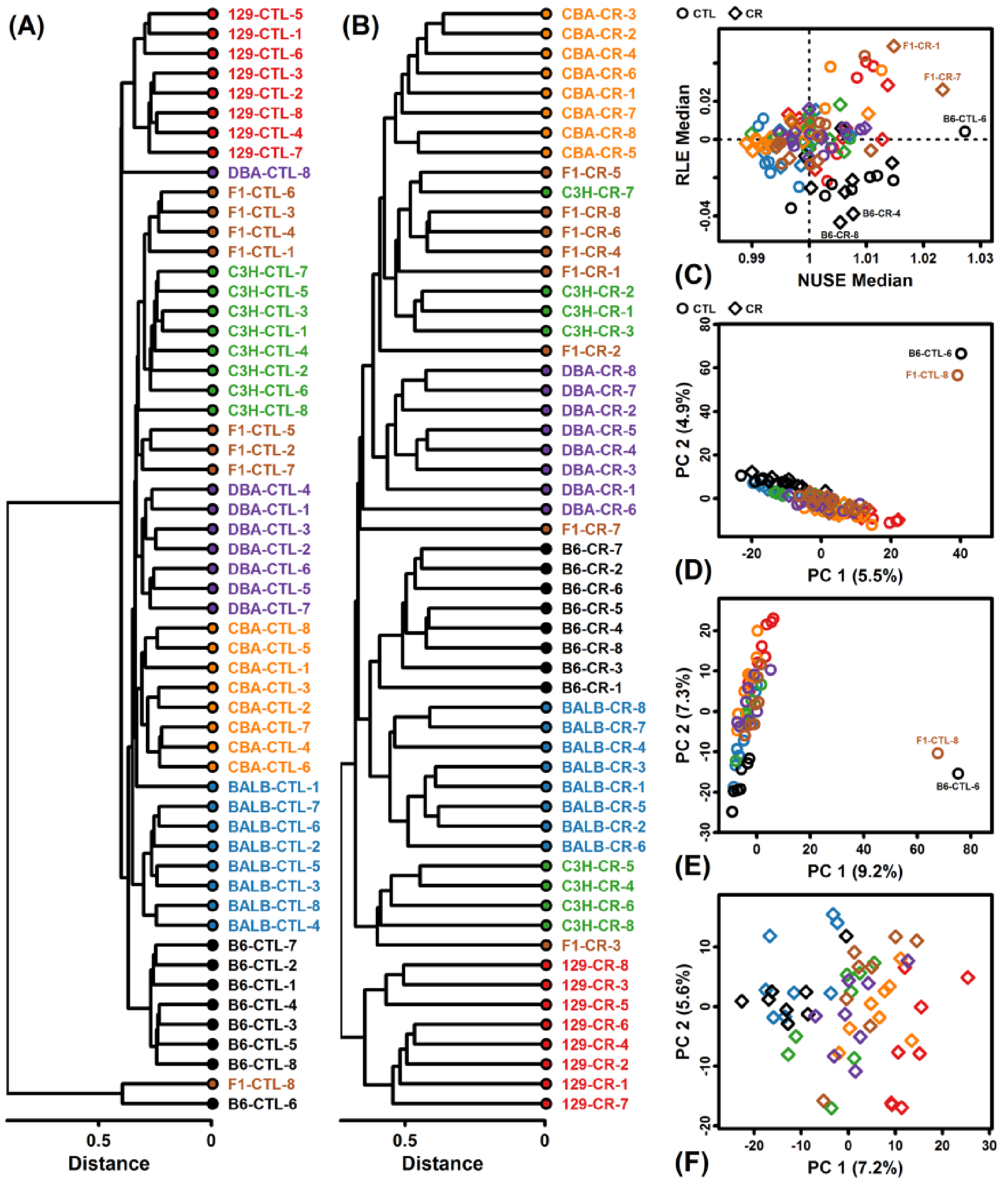

Figure S15. Cortex sample quality control (n = 112 samples). (**A**) CTL sample cluster analysis. (**B**) CR sample cluster analysis. In (A) and (B), samples were clustered based upon the Euclidean distance between expression profiles of protein-coding genes (average linkage method). (**C**) Scatterplot comparison between probe-level model NUSE and RLE medians (NUSE: normalized unscaled standard error; RLE: relative log expression). Sample hybridizations with NUSE median differing substantially from 1 or RLE median differing substantially from 0 are potentially of low quality. (**D**) PC scatterplot (CTL + CR samples). (**E**) PC scatterplot (CTL samples only). (**F**) PC scatterplot (CR samples only).

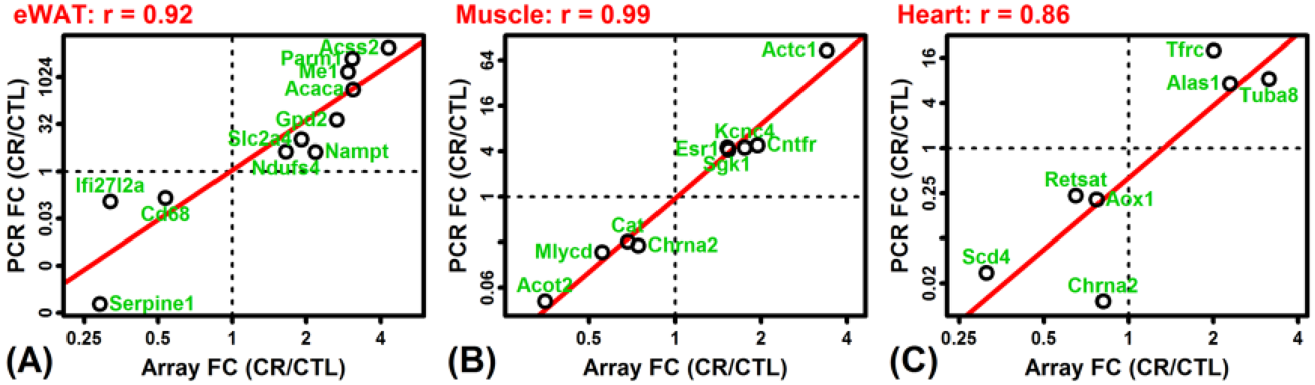

Figure S16. Comparison of FC estimates (CR/CTL) between microarray and RT-PCR (C57BL/6). Microarray and RT-PCR FC estimates were compared for selected genes with respect to (**A**) eWAT, (**B**) muscle and (**C**) heart. The least squares regression estimate is shown (red line) with Pearson correlation coefficient (top margin). RT-PCR estimates were obtained from Table S6 in Barger et al. 2017 (Aging Cell 16:750-760).

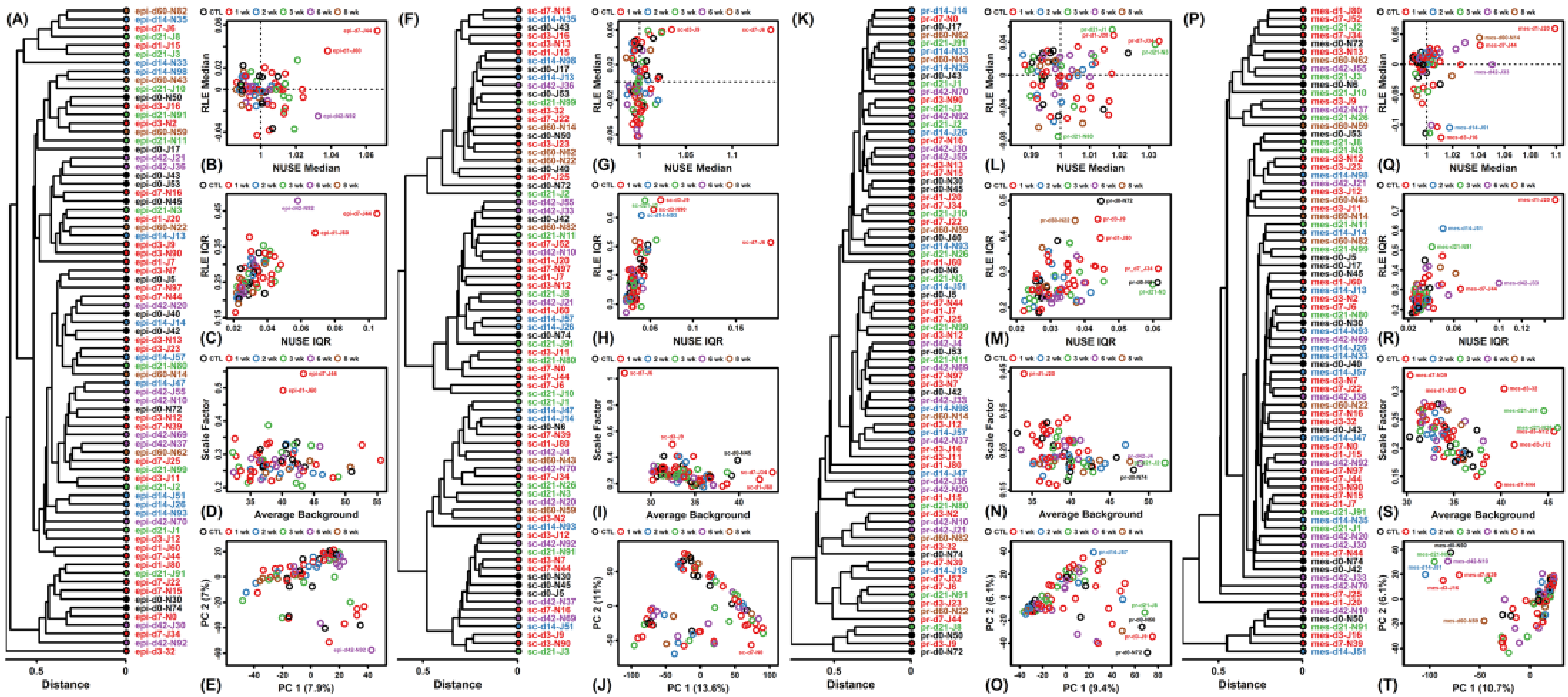

Figure S17. Quality control assessments for microarray analysis of multiple WAT depots in *ad lib* and CR-fed obese C57BL/6 mice (GSE30534; *n* = 312 samples). Quality control results are shown for (**A – E**) epididymal, (**F – J**) subcutaneous, (**K – O**) perirenal, and (**P – T**) mesenteric WAT samples. (A, F, K, P) Cluster analyses. Samples were clustered based upon the Euclidean distance between expression profiles of protein-coding genes (average linkage method). (B, G, L, Q) Scatterplot comparisons between probe-level model NUSE and RLE medians (NUSE: normalized unscaled standard error; RLE: relative log expression). Sample hybridizations with NUSE median differing substantially from 1 or RLE median differing substantially from 0 are potentially of low quality. (C, H, M, R) Scatterplot comparisons between probe-level model NUSE and RLE interquartile range (IQR). Sample hybridizations with increased IQR estimates are potentially of low quality. (D, I, N, S) Scatterplot comparisons between average background and scale factor. Sample hybridizations with extreme differences in background and/or scale factor are less reliably compared under standard array normalization protocols (e.g., robust multichip average, RMA). (E, J, O, T) Principal component scatterplots.
